# Supplementary material for: Exploring the Impact of an Innovative Peer Role-Play Simulation to Cultivate Student Pharmacists’ Motivational Interviewing Skills
Source: Pharmacy (Basel). 2023 Jul 29;11(4):122. doi: 10.3390/pharmacy11040122 (PMC10458726; doi:10.3390/pharmacy11040122)
Supplement: Supplementary file 1 [file pharmacy-11-00122-s001.zip › Document S1-Asthma and Medication Primer.pdf]

Asthma Primer

PHM 318: Foundations of Pharmacy

This primer accompanies the *Motivational Interviewing – Role Play Instructions and Timeline* PowerPoint used for the Motivational Interviewing Role Play (Video Submission) assignment.

Overview

This primer provides an overview of asthma as a disease state, as well as two **fictional** medications used to treat asthma: **Alpha** and **Omega**. Although Alpha and Omega are loosely based on actual maintenance and emergency (rescue) medications and reflect some principles that are important in asthma counseling, please remember that these medications have been fictionalized.

Most of you will learn a great deal about asthma medications before you graduate, but as a P1 student in Foundations of Pharmacy, you likely do not have the expertise yet to counsel patients about them in detail. Rather than risk having you practice giving out inaccurate or incomplete information about real medications, we will use Alpha and Omega as "stand-ins." This is perfectly suited to an assignment in which we want you to focus on **communication skills**, rather than knowledge of therapeutics. Moreover, the purpose of Motivational Interviewing is to enhance patients' intrinsic motivation, not to provide extensive patient education.

What is Asthma?

Asthma is a disorder that causes inflammation in the lungs and/or airways. This inflammation can cause irritation that creates feelings of tightness or constriction in the lungs. Early asthma research focused on bronchial spasms, genetics, and triggers like allergens, extreme emotion, and diet. Most patients used smooth muscle relaxers called bronchodilators or rescue inhalers. This was the standard of care until the 1960-80s, when overuse of these agents, and an increase in overall allergies, led to a significant increase in death. This generated more research and a better understanding of asthma as an inflammatory and immune system issue.

The most recent guidelines changed significantly and are now focused on using controllers extensively. You will learn about controllers and relievers in your second and third professional years. For now, we have provided you with all the information you need on two **fictional** medications that are appropriate for your patient.

For more information, visit: https://www.asthma.com

Medication Alpha

Alpha is a **maintenance (preventive) medication** designed to be taken daily to reduce the occurrence of asthma symptoms.

Indication (FDA)	Asthma
Dosing	Two oral inhalations twice daily (AM and PM)
Administration	Prime the inhaler (2 sprays), then shake well, using a long deep breath to get spray into your lungs. After use, rinse out mouth.
Contraindications	Hypersensitivity (allergic reaction) to Alpha; Not a rescue inhaler
How supplied	Inhalation of Aerosol Liquid

Medication Omega

Omega is a **rescue medication** designed to be taken only when a person is experiencing asthma symptoms.

Indication (FDA)	Asthma
Dosing	Two oral inhalations every 4-6 hours as needed for shortness of breath
Administration	Prime the inhaler (2 sprays), then shake well, using long deep breath to get spray into your lungs. After use, rinse out mouth.
Contraindications:	Hypersensitivity (allergic reaction) to Omega
How supplied:	Inhalation of Aerosol Liquid

Glossary

Term	*Definition*
Aerosol liquid	A suspension of fine liquid droplets in air
Inhalations	The act of inhaling, breathing in air

Bronchospasm	An abnormal contraction of the bronchi (large airway in your lungs) resulting in constriction and difficulty breathing
Bronchodilator	A drug that widens the air passages of the lungs by relaxing bronchial smooth muscle
Inflammation	A localized protective reaction of tissue to irritation, injury, or infection; identified by pain, redness, swelling, or loss of function
