# Supplementary material for: Exploring the Impact of an Innovative Peer Role-Play Simulation to Cultivate Student Pharmacists’ Motivational Interviewing Skills
Source: Pharmacy (Basel). 2023 Jul 29;11(4):122. doi: 10.3390/pharmacy11040122 (PMC10458726; doi:10.3390/pharmacy11040122)
Supplement: Supplementary file 1 [file pharmacy-11-00122-s001.zip › PowerPoint Slides S1-Turn-by-turn action script .pptx]

## Slide 1
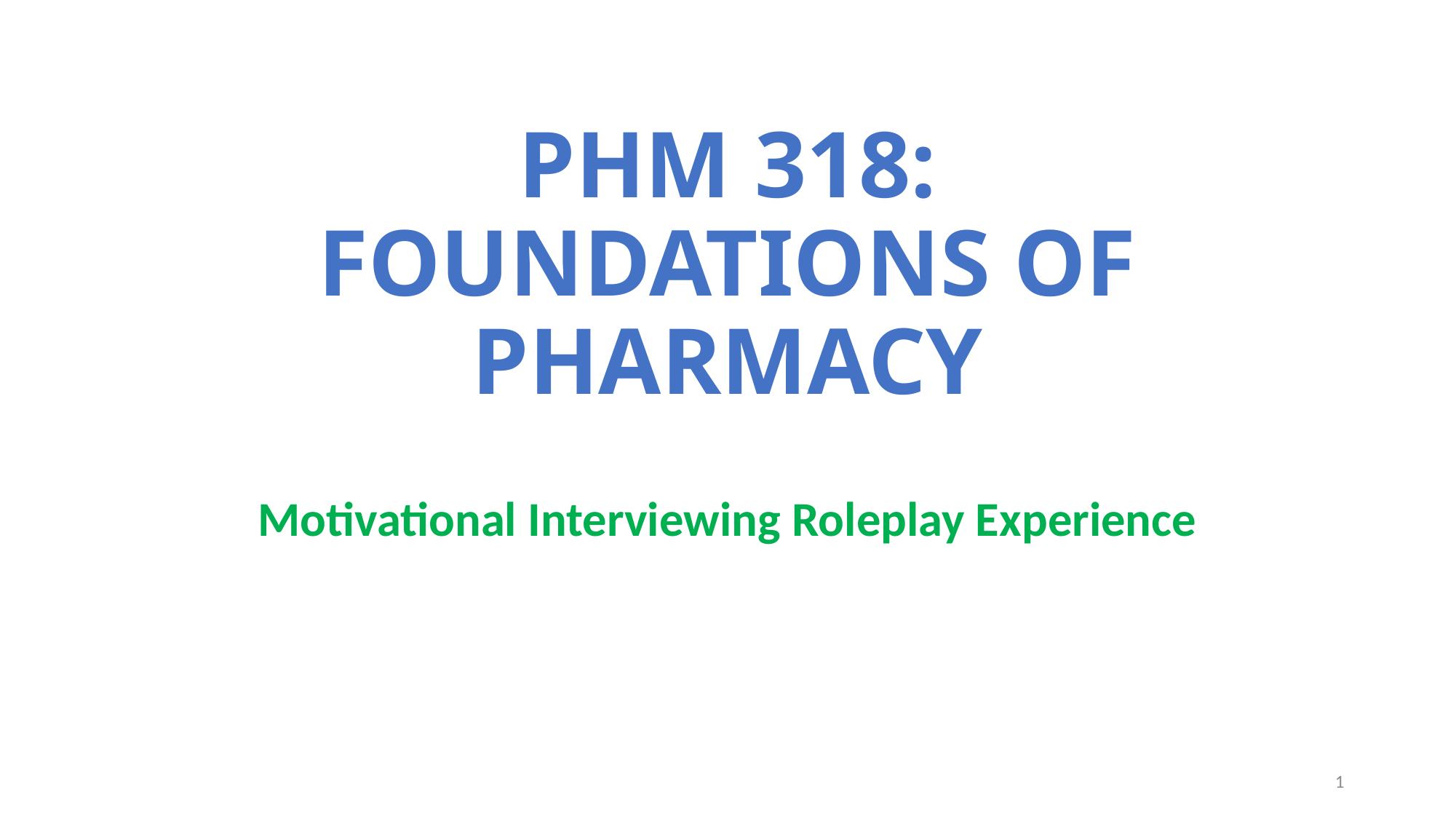

# PHM 318: FOUNDATIONS OF PHARMACY
Motivational Interviewing Roleplay Experience
1

## Slide 2
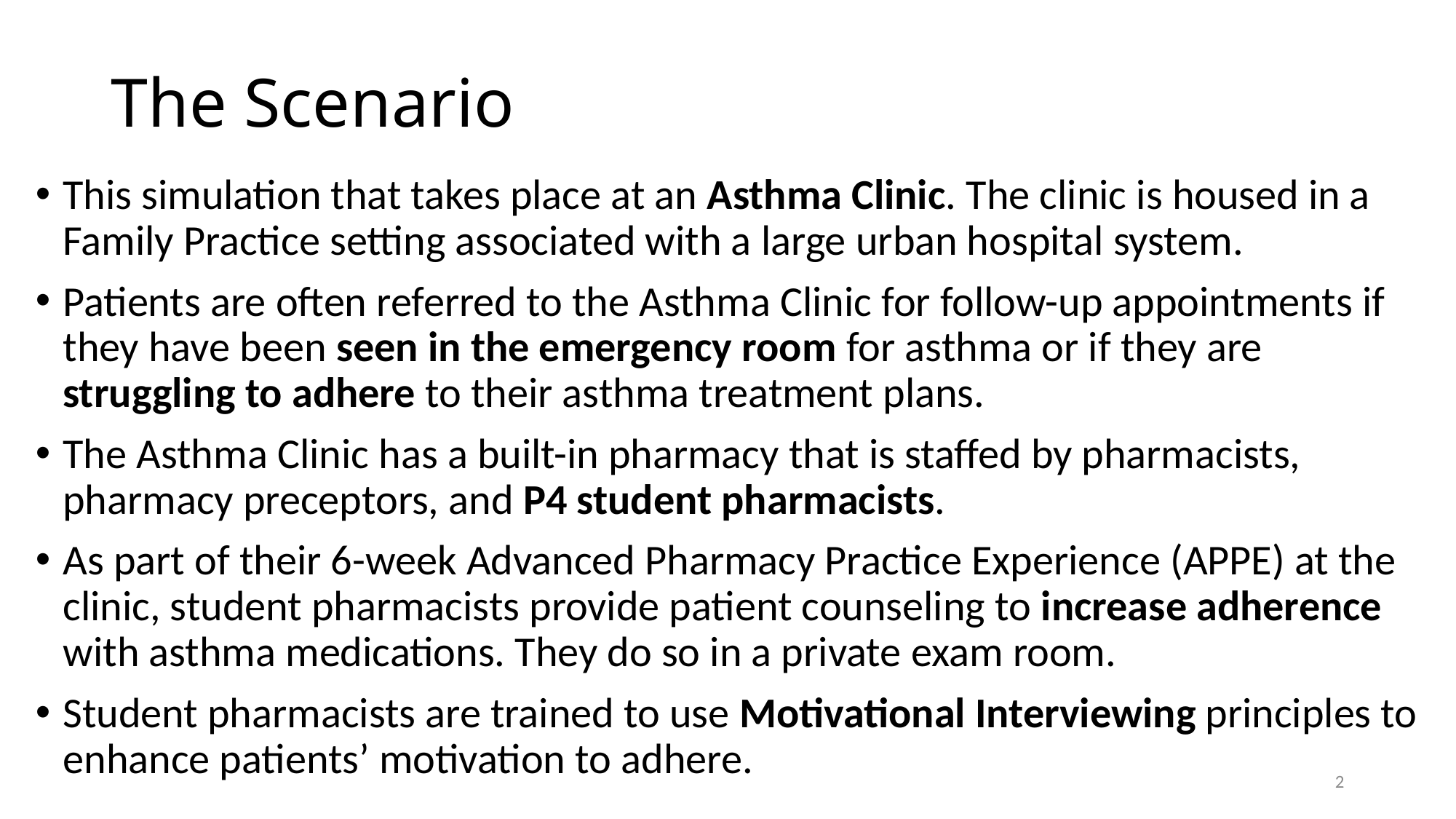

# The Scenario
This simulation that takes place at an Asthma Clinic. The clinic is housed in a Family Practice setting associated with a large urban hospital system.
Patients are often referred to the Asthma Clinic for follow-up appointments if they have been seen in the emergency room for asthma or if they are struggling to adhere to their asthma treatment plans.
The Asthma Clinic has a built-in pharmacy that is staffed by pharmacists, pharmacy preceptors, and P4 student pharmacists.
As part of their 6-week Advanced Pharmacy Practice Experience (APPE) at the clinic, student pharmacists provide patient counseling to increase adherence with asthma medications. They do so in a private exam room.
Student pharmacists are trained to use Motivational Interviewing principles to enhance patients’ motivation to adhere.
2

## Slide 3
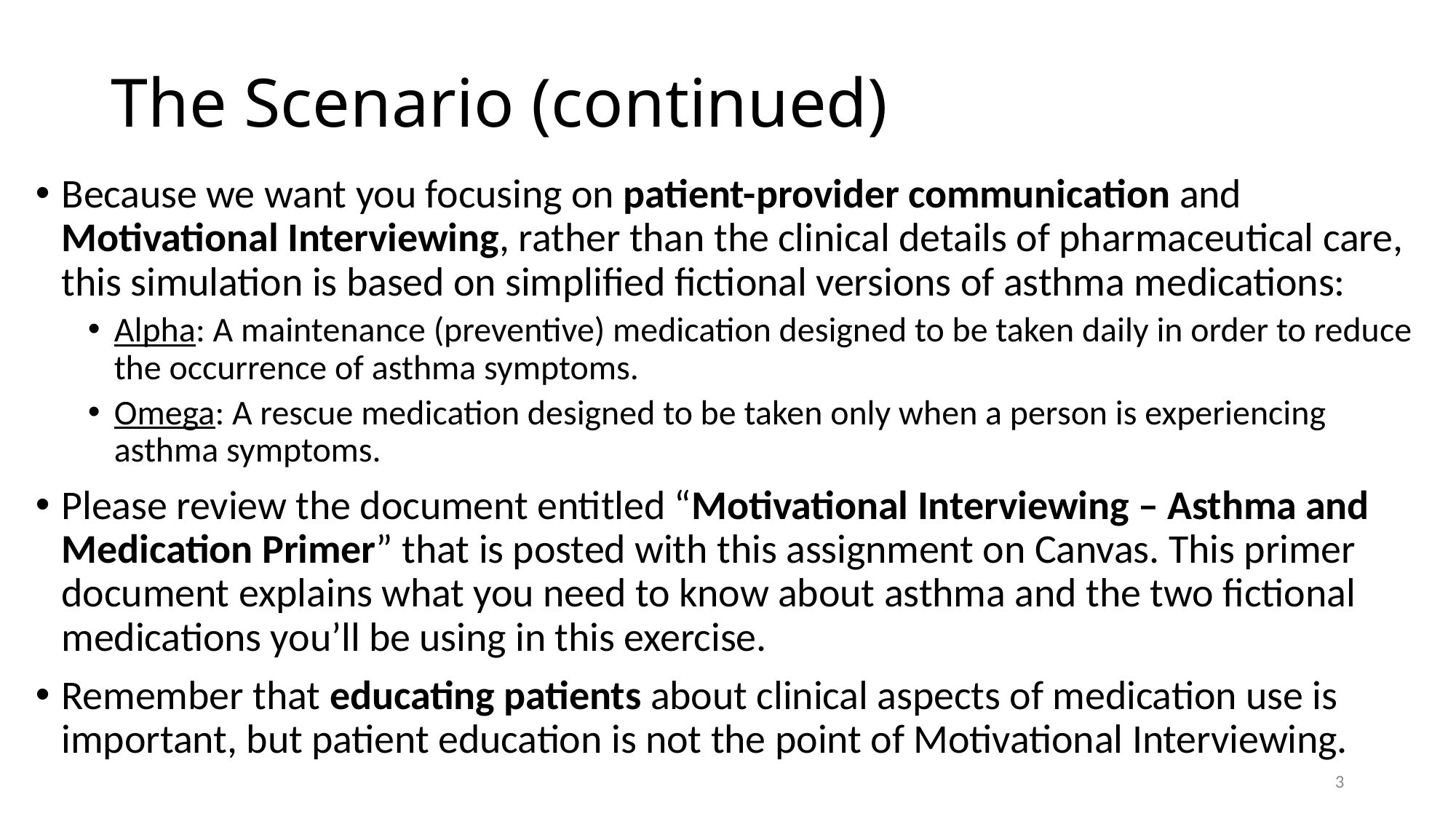

# The Scenario (continued)
Because we want you focusing on patient-provider communication and Motivational Interviewing, rather than the clinical details of pharmaceutical care, this simulation is based on simplified fictional versions of asthma medications:
Alpha: A maintenance (preventive) medication designed to be taken daily in order to reduce the occurrence of asthma symptoms.
Omega: A rescue medication designed to be taken only when a person is experiencing asthma symptoms.
Please review the document entitled “Motivational Interviewing – Asthma and Medication Primer” that is posted with this assignment on Canvas. This primer document explains what you need to know about asthma and the two fictional medications you’ll be using in this exercise.
Remember that educating patients about clinical aspects of medication use is important, but patient education is not the point of Motivational Interviewing.
3

## Slide 4
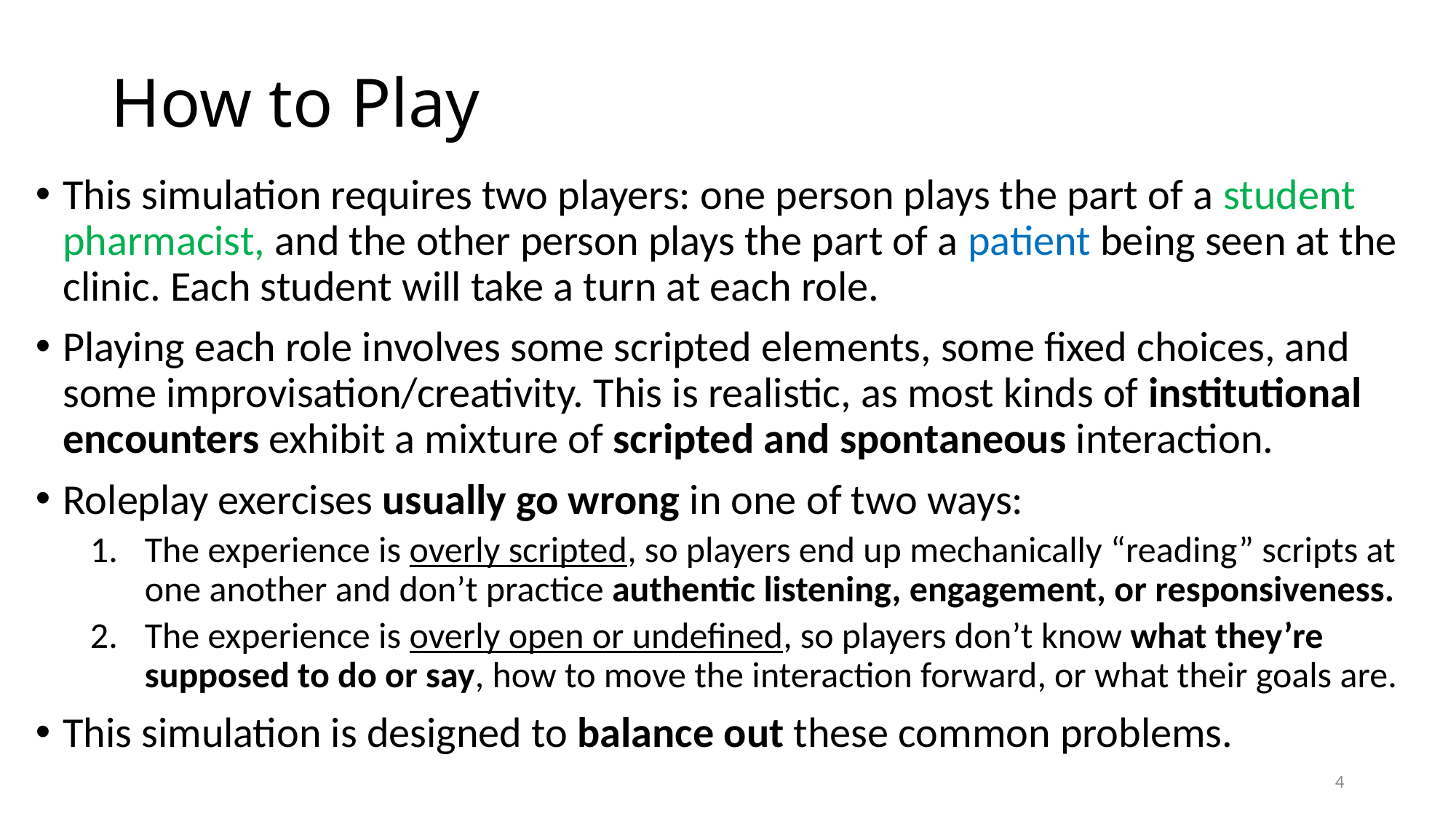

# How to Play
This simulation requires two players: one person plays the part of a student pharmacist, and the other person plays the part of a patient being seen at the clinic. Each student will take a turn at each role.
Playing each role involves some scripted elements, some fixed choices, and some improvisation/creativity. This is realistic, as most kinds of institutional encounters exhibit a mixture of scripted and spontaneous interaction.
Roleplay exercises usually go wrong in one of two ways:
The experience is overly scripted, so players end up mechanically “reading” scripts at one another and don’t practice authentic listening, engagement, or responsiveness.
The experience is overly open or undefined, so players don’t know what they’re supposed to do or say, how to move the interaction forward, or what their goals are.
This simulation is designed to balance out these common problems.
4

## Slide 5
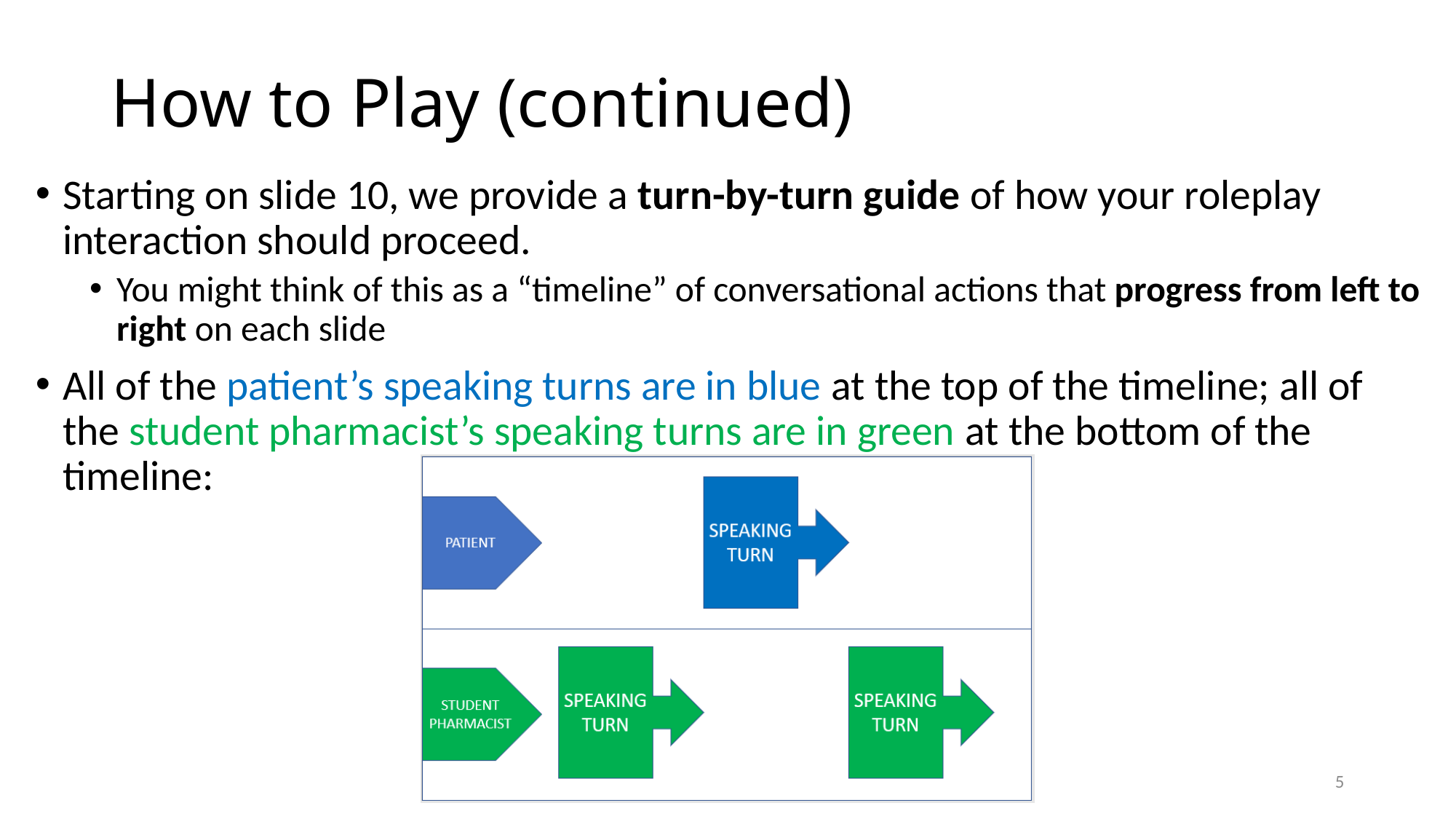

# How to Play (continued)
Starting on slide 10, we provide a turn-by-turn guide of how your roleplay interaction should proceed.
You might think of this as a “timeline” of conversational actions that progress from left to right on each slide
All of the patient’s speaking turns are in blue at the top of the timeline; all of the student pharmacist’s speaking turns are in green at the bottom of the timeline:
5

## Slide 6
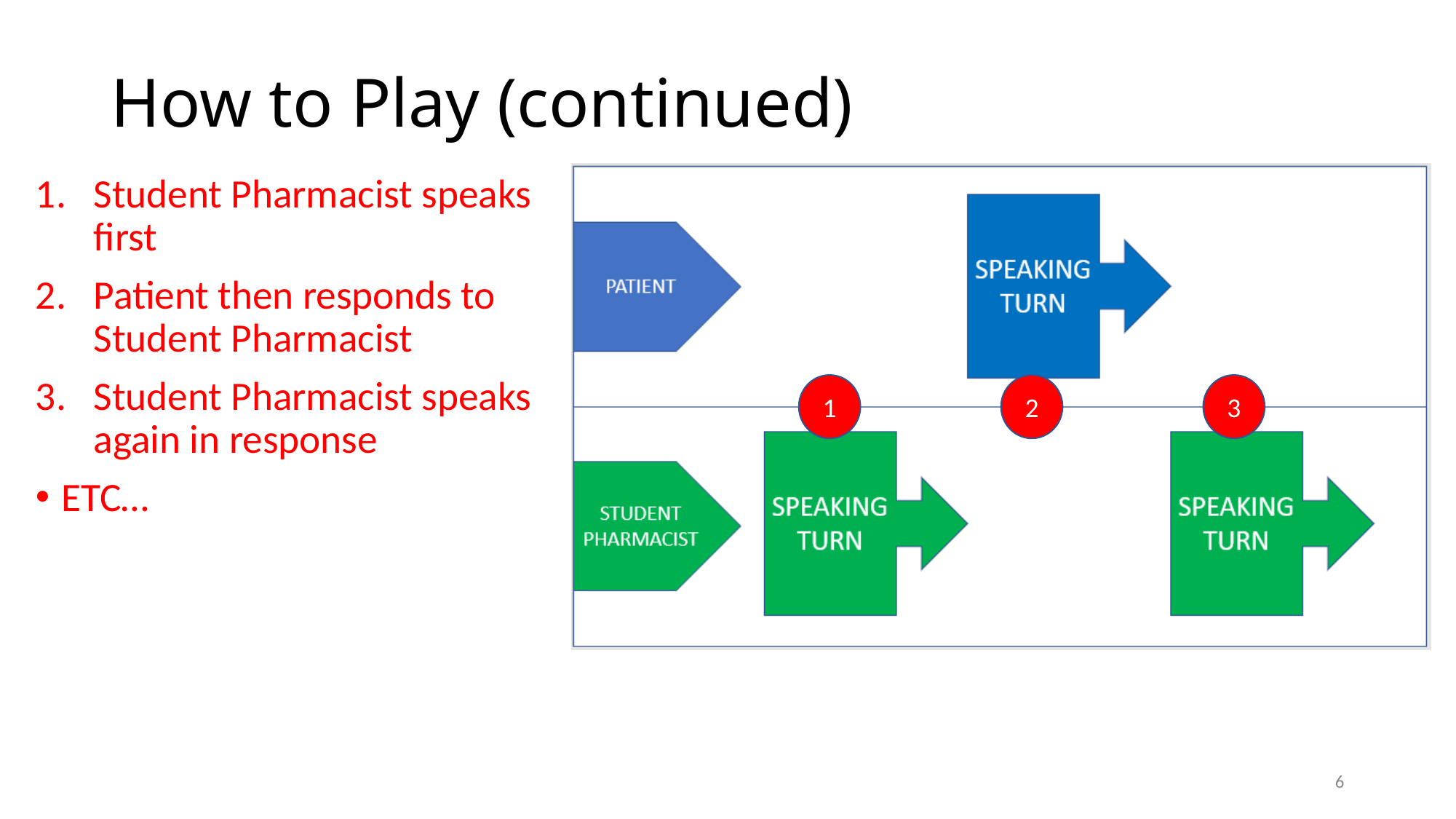

# How to Play (continued)
Student Pharmacist speaks first
Patient then responds to Student Pharmacist
Student Pharmacist speaks again in response
ETC…
1
2
3
6

## Slide 7
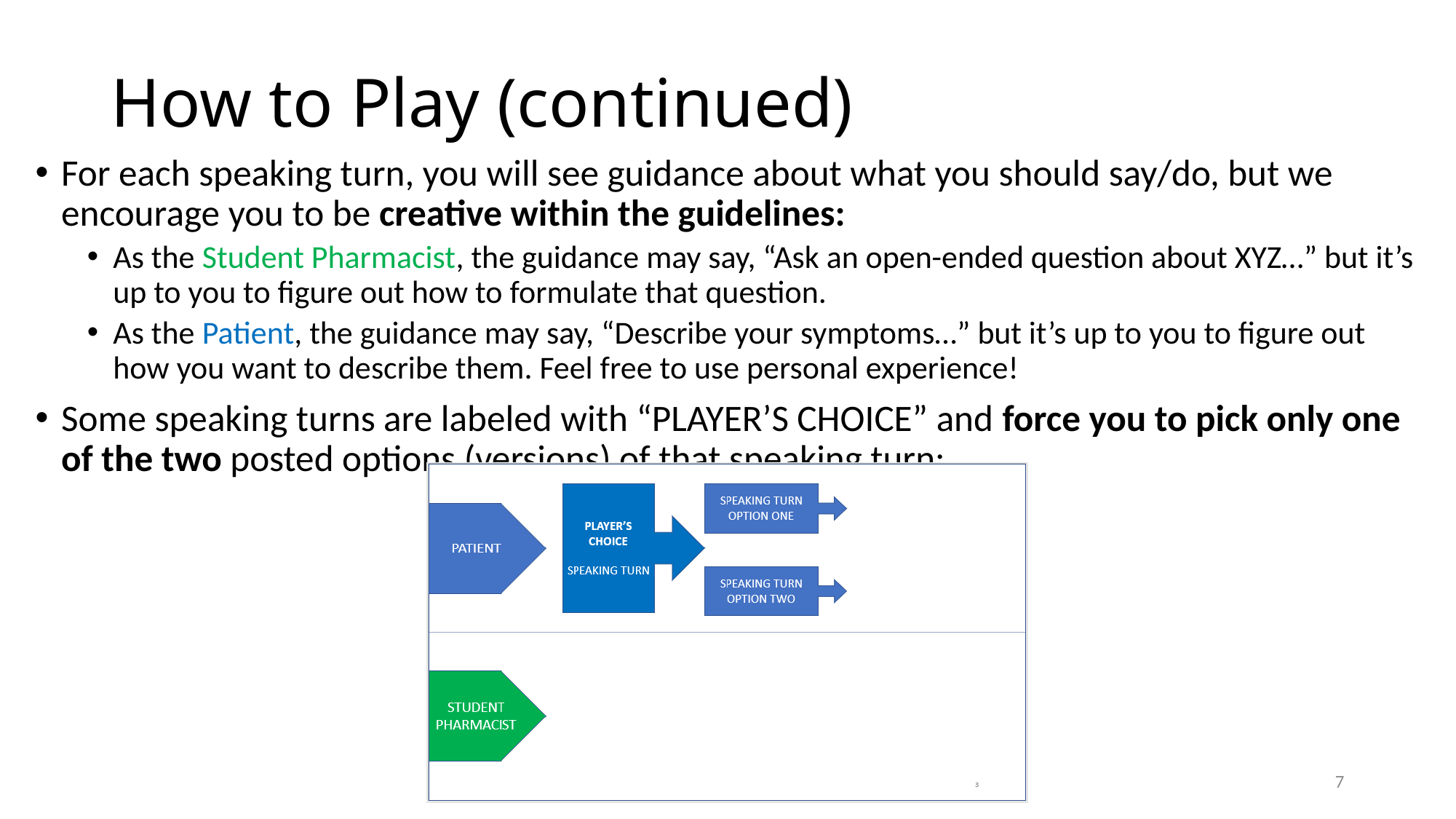

# How to Play (continued)
For each speaking turn, you will see guidance about what you should say/do, but we encourage you to be creative within the guidelines:
As the Student Pharmacist, the guidance may say, “Ask an open-ended question about XYZ…” but it’s up to you to figure out how to formulate that question.
As the Patient, the guidance may say, “Describe your symptoms…” but it’s up to you to figure out how you want to describe them. Feel free to use personal experience!
Some speaking turns are labeled with “PLAYER’S CHOICE” and force you to pick only one of the two posted options (versions) of that speaking turn:
7

## Slide 8
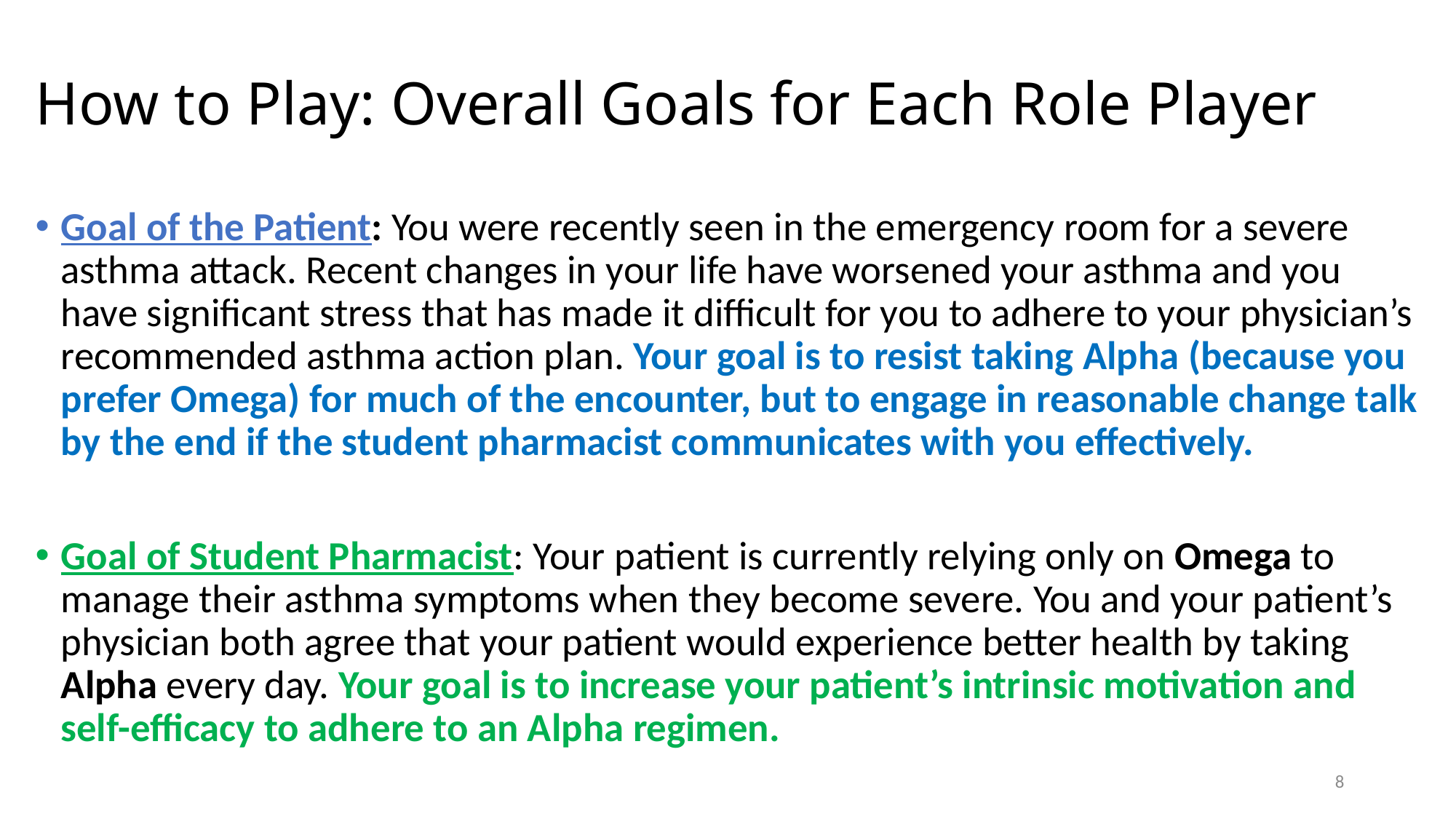

# How to Play: Overall Goals for Each Role Player
Goal of the Patient: You were recently seen in the emergency room for a severe asthma attack. Recent changes in your life have worsened your asthma and you have significant stress that has made it difficult for you to adhere to your physician’s recommended asthma action plan. Your goal is to resist taking Alpha (because you prefer Omega) for much of the encounter, but to engage in reasonable change talk by the end if the student pharmacist communicates with you effectively.
Goal of Student Pharmacist: Your patient is currently relying only on Omega to manage their asthma symptoms when they become severe. You and your patient’s physician both agree that your patient would experience better health by taking Alpha every day. Your goal is to increase your patient’s intrinsic motivation and self-efficacy to adhere to an Alpha regimen.
8

## Slide 9
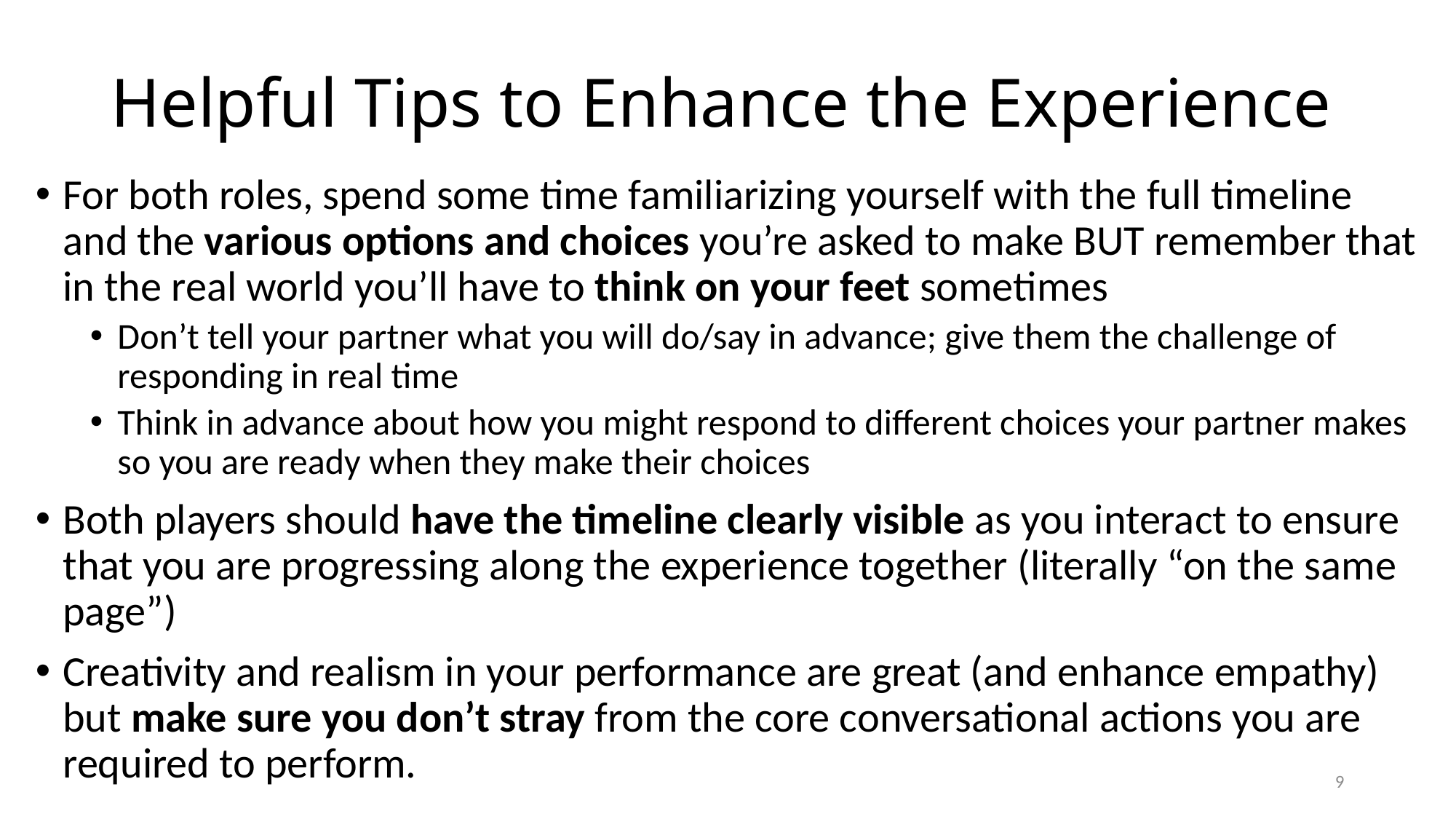

# Helpful Tips to Enhance the Experience
For both roles, spend some time familiarizing yourself with the full timeline and the various options and choices you’re asked to make BUT remember that in the real world you’ll have to think on your feet sometimes
Don’t tell your partner what you will do/say in advance; give them the challenge of responding in real time
Think in advance about how you might respond to different choices your partner makes so you are ready when they make their choices
Both players should have the timeline clearly visible as you interact to ensure that you are progressing along the experience together (literally “on the same page”)
Creativity and realism in your performance are great (and enhance empathy) but make sure you don’t stray from the core conversational actions you are required to perform.
9

## Slide 10
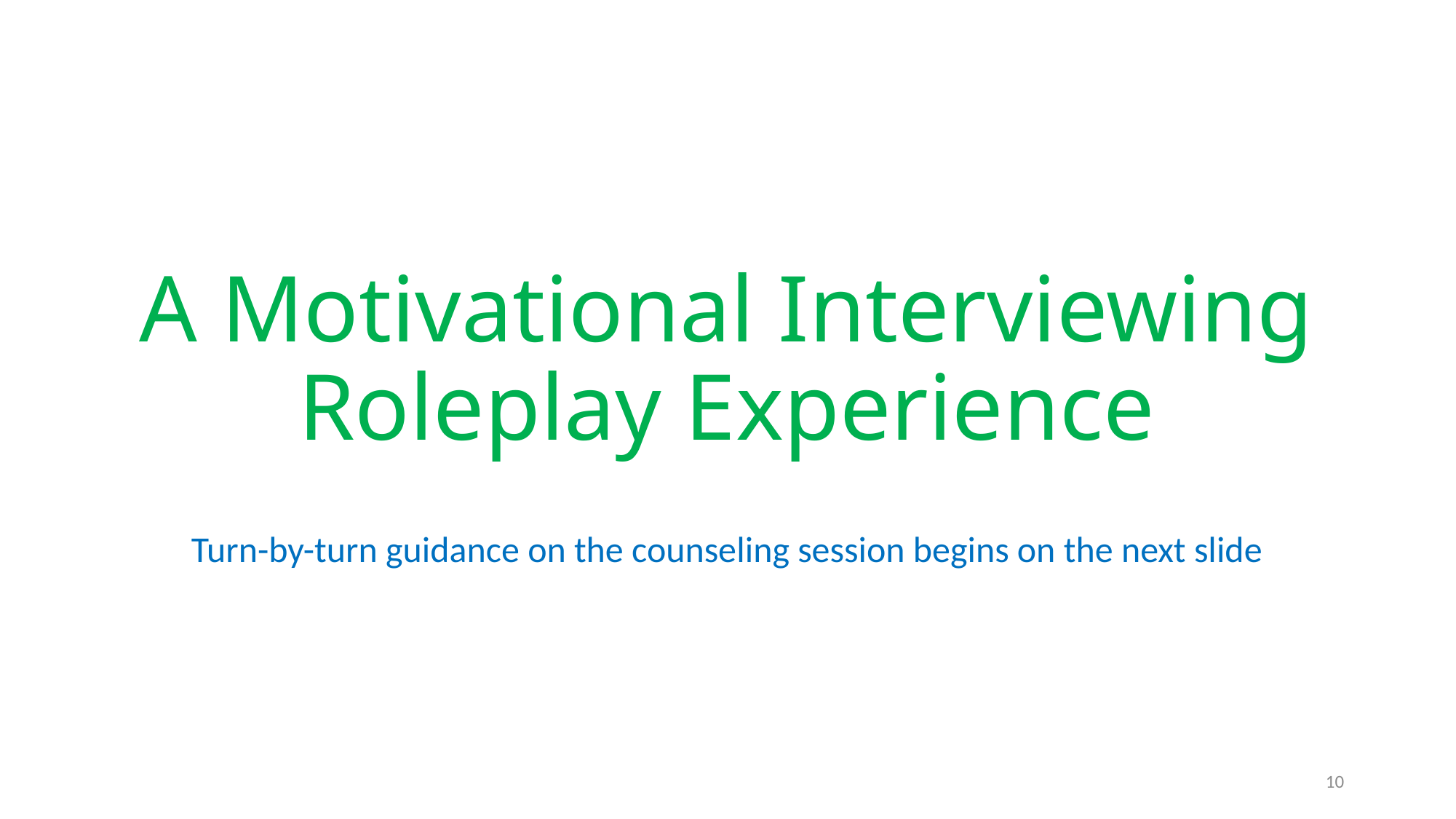

# A Motivational Interviewing Roleplay Experience
Turn-by-turn guidance on the counseling session begins on the next slide
10

## Slide 11
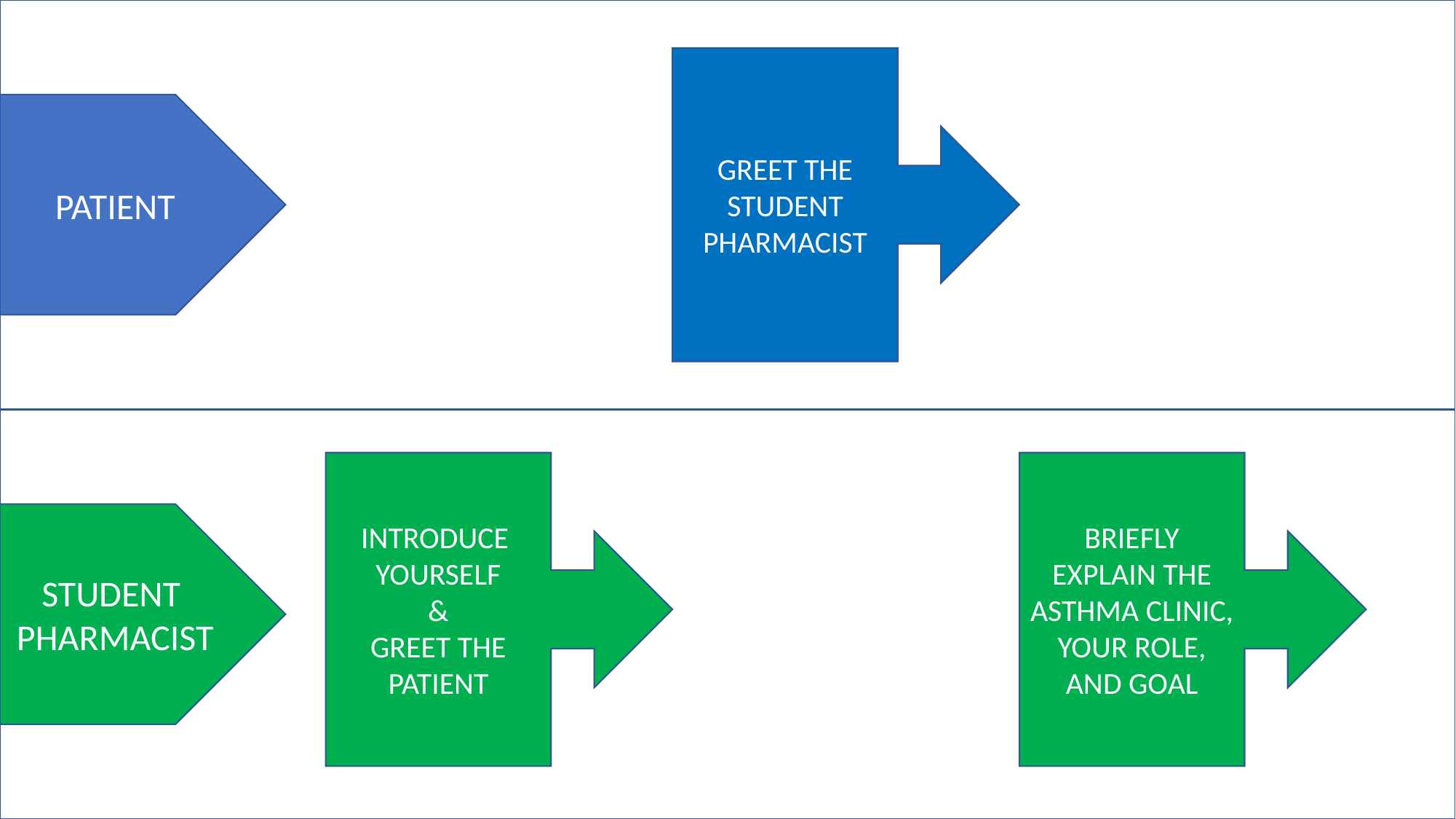

GREET THE STUDENT PHARMACIST
PATIENT
BRIEFLY EXPLAIN THE ASTHMA CLINIC, YOUR ROLE, AND GOAL
INTRODUCE
YOURSELF
&
GREET THE PATIENT
STUDENT
PHARMACIST
11

## Slide 12
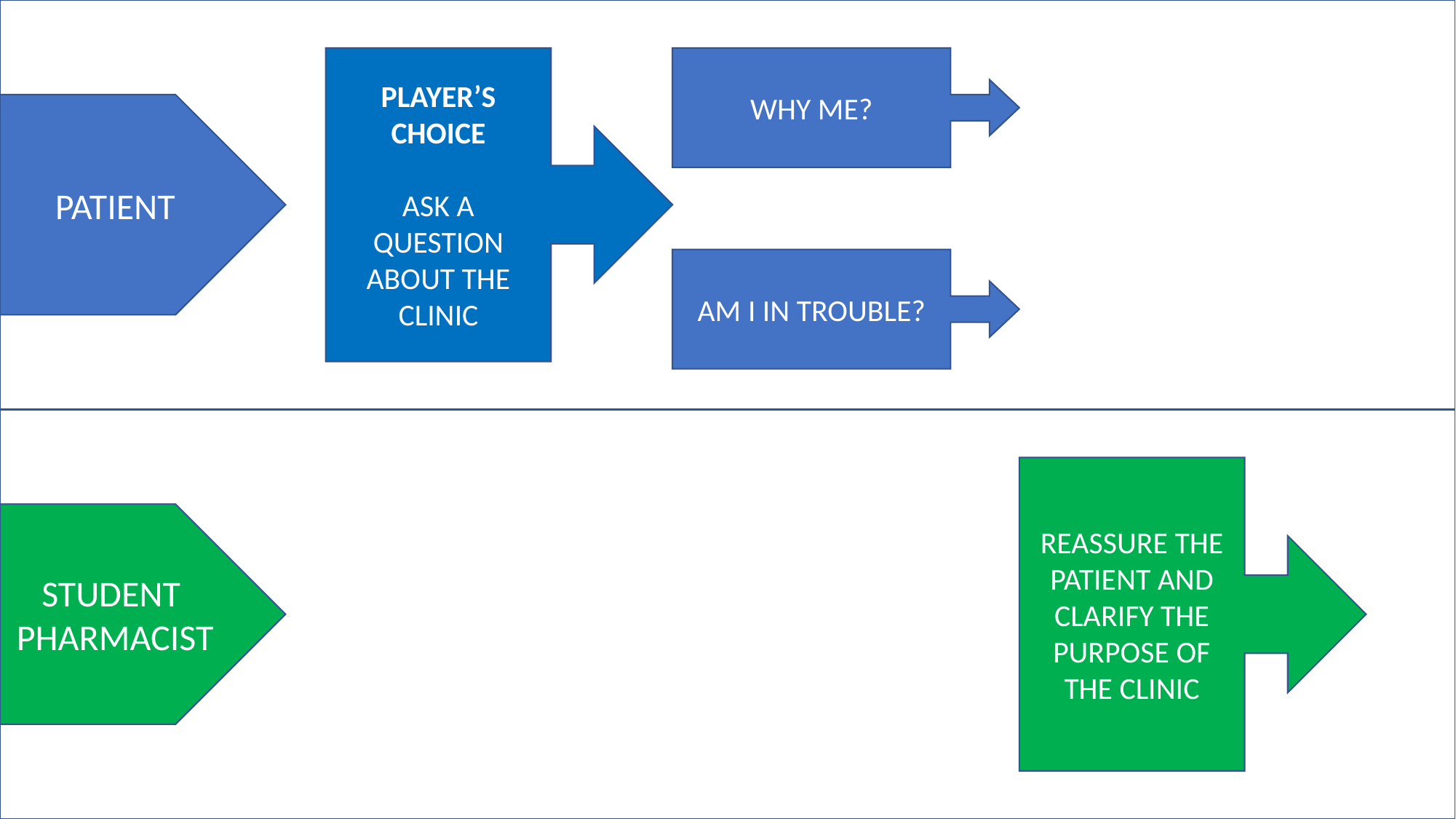

PLAYER’S CHOICE
ASK A QUESTION ABOUT THE CLINIC
WHY ME?
PATIENT
AM I IN TROUBLE?
REASSURE THE PATIENT AND CLARIFY THE PURPOSE OF THE CLINIC
STUDENT
PHARMACIST
12

## Slide 13
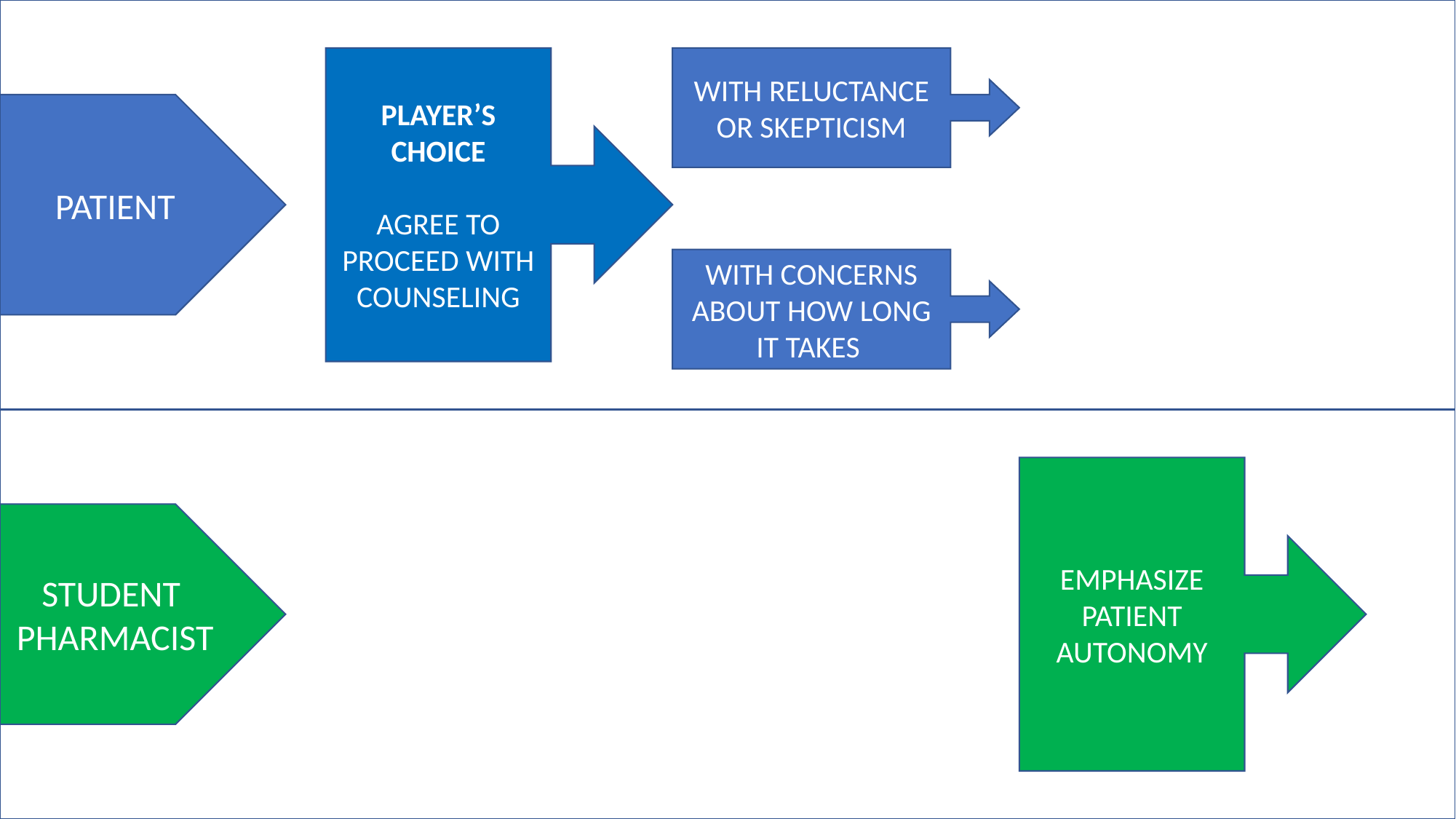

PLAYER’S CHOICE
AGREE TO PROCEED WITH COUNSELING
WITH RELUCTANCE
OR SKEPTICISM
PATIENT
WITH CONCERNS ABOUT HOW LONG IT TAKES
EMPHASIZE PATIENT AUTONOMY
STUDENT
PHARMACIST
13

## Slide 14
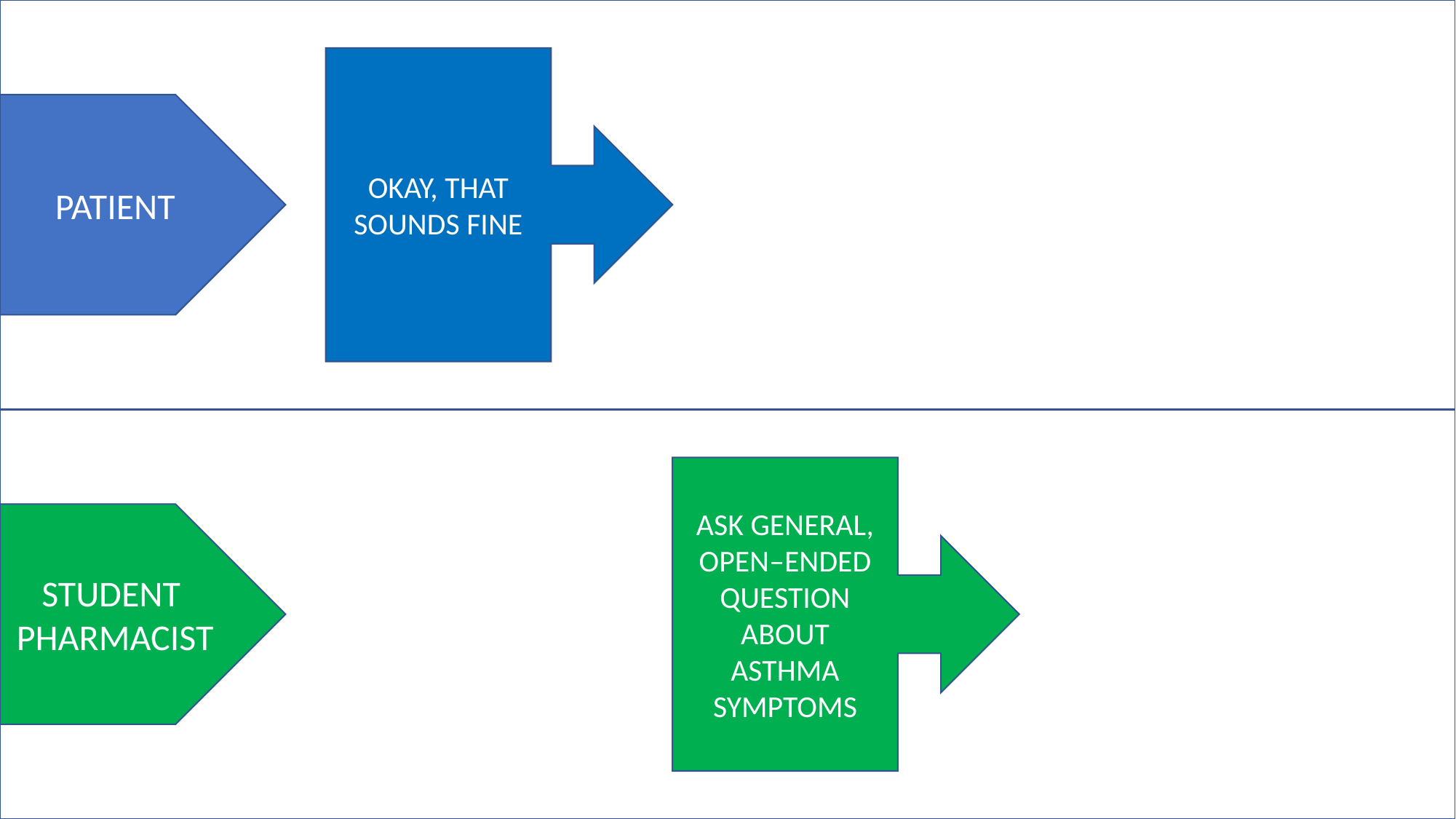

OKAY, THAT SOUNDS FINE
PATIENT
ASK GENERAL, OPEN–ENDED QUESTION ABOUT ASTHMA SYMPTOMS
STUDENT
PHARMACIST
14

## Slide 15
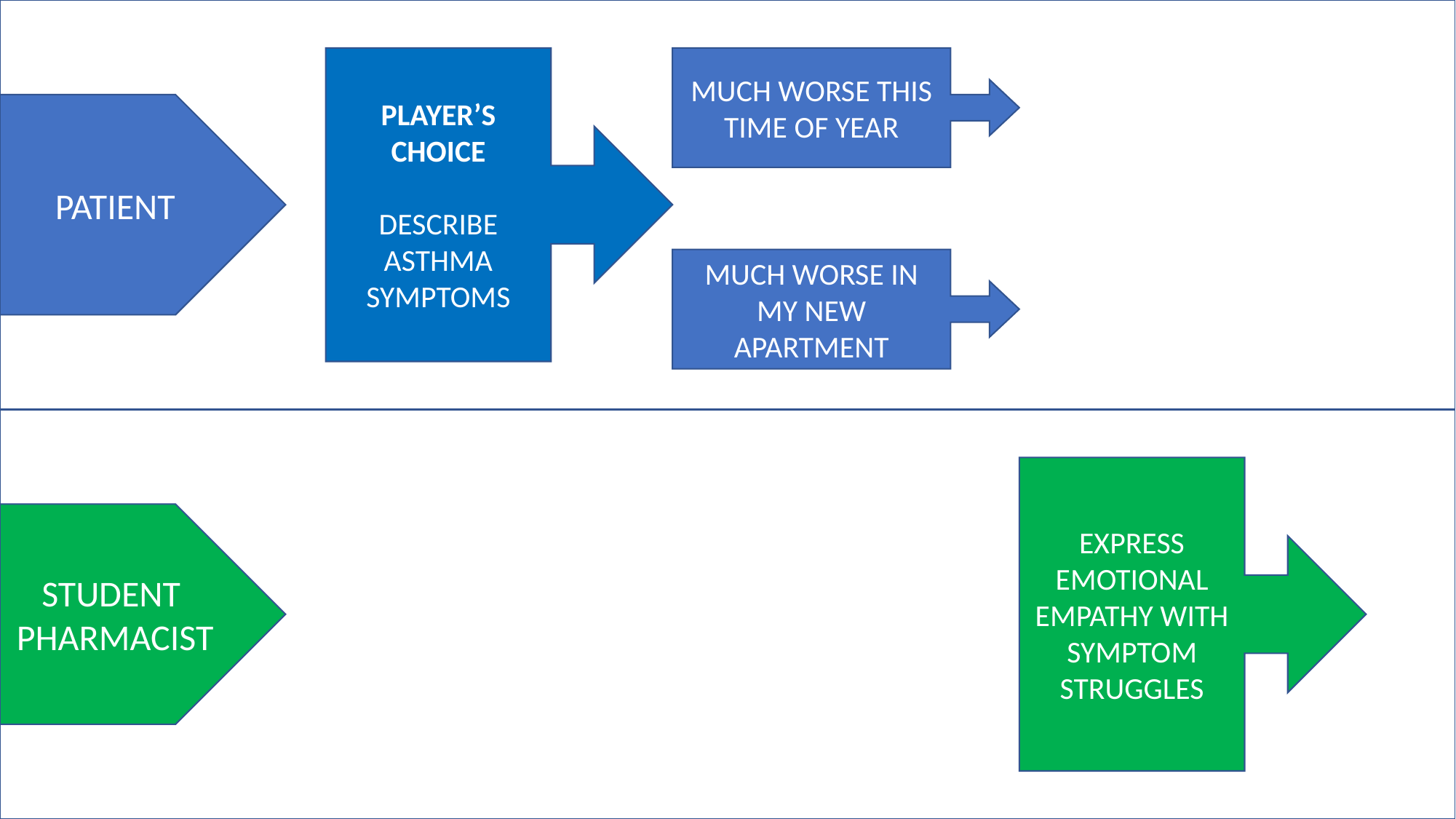

PLAYER’S CHOICE
DESCRIBE ASTHMA SYMPTOMS
MUCH WORSE THIS TIME OF YEAR
PATIENT
MUCH WORSE IN MY NEW APARTMENT
EXPRESS
EMOTIONAL EMPATHY WITH
SYMPTOM STRUGGLES
STUDENT
PHARMACIST
15

## Slide 16
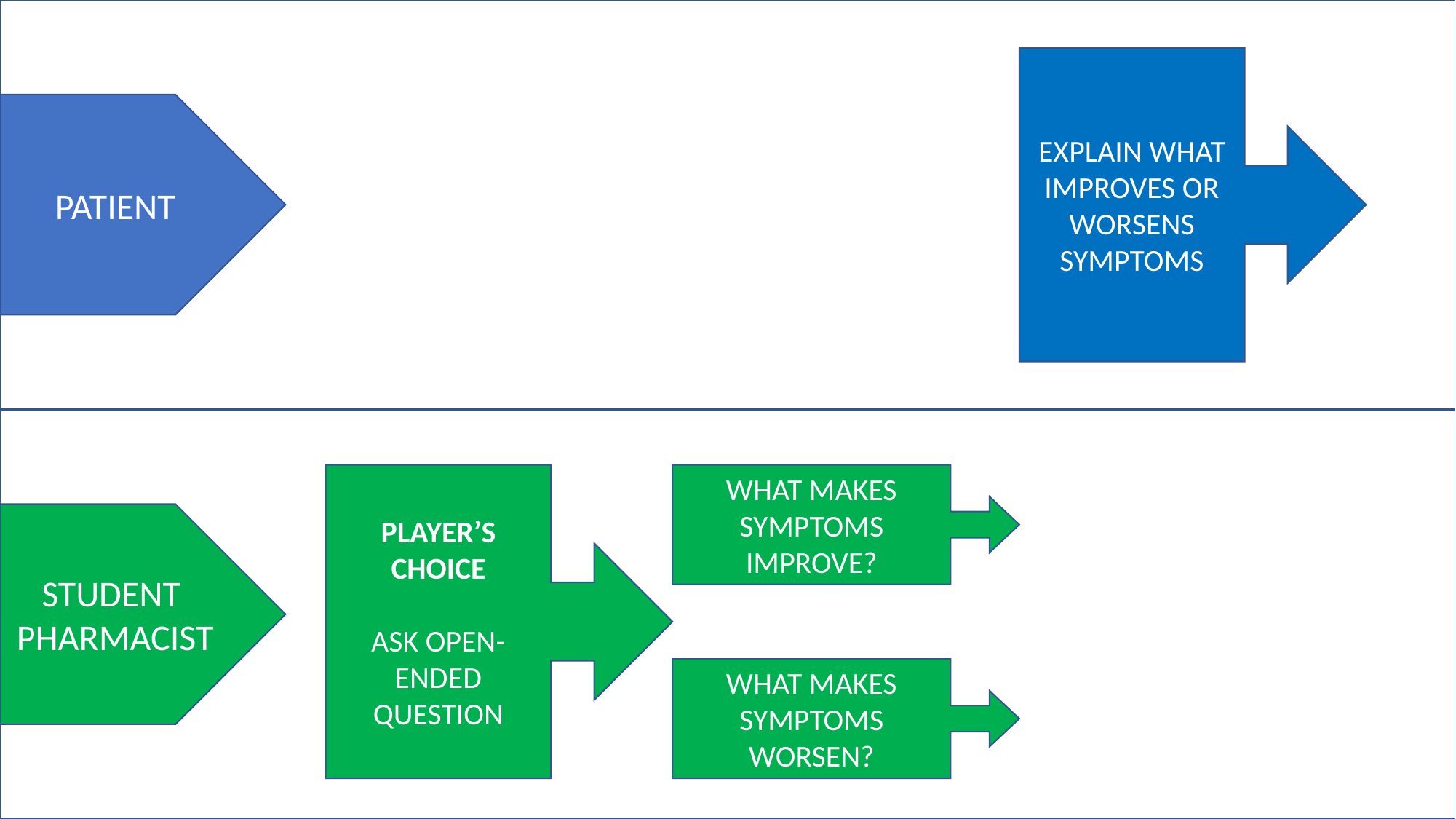

EXPLAIN WHAT IMPROVES OR WORSENS SYMPTOMS
PATIENT
PLAYER’S CHOICE
ASK OPEN-ENDED QUESTION
WHAT MAKES SYMPTOMS IMPROVE?
STUDENT
PHARMACIST
WHAT MAKES SYMPTOMS WORSEN?
16

## Slide 17
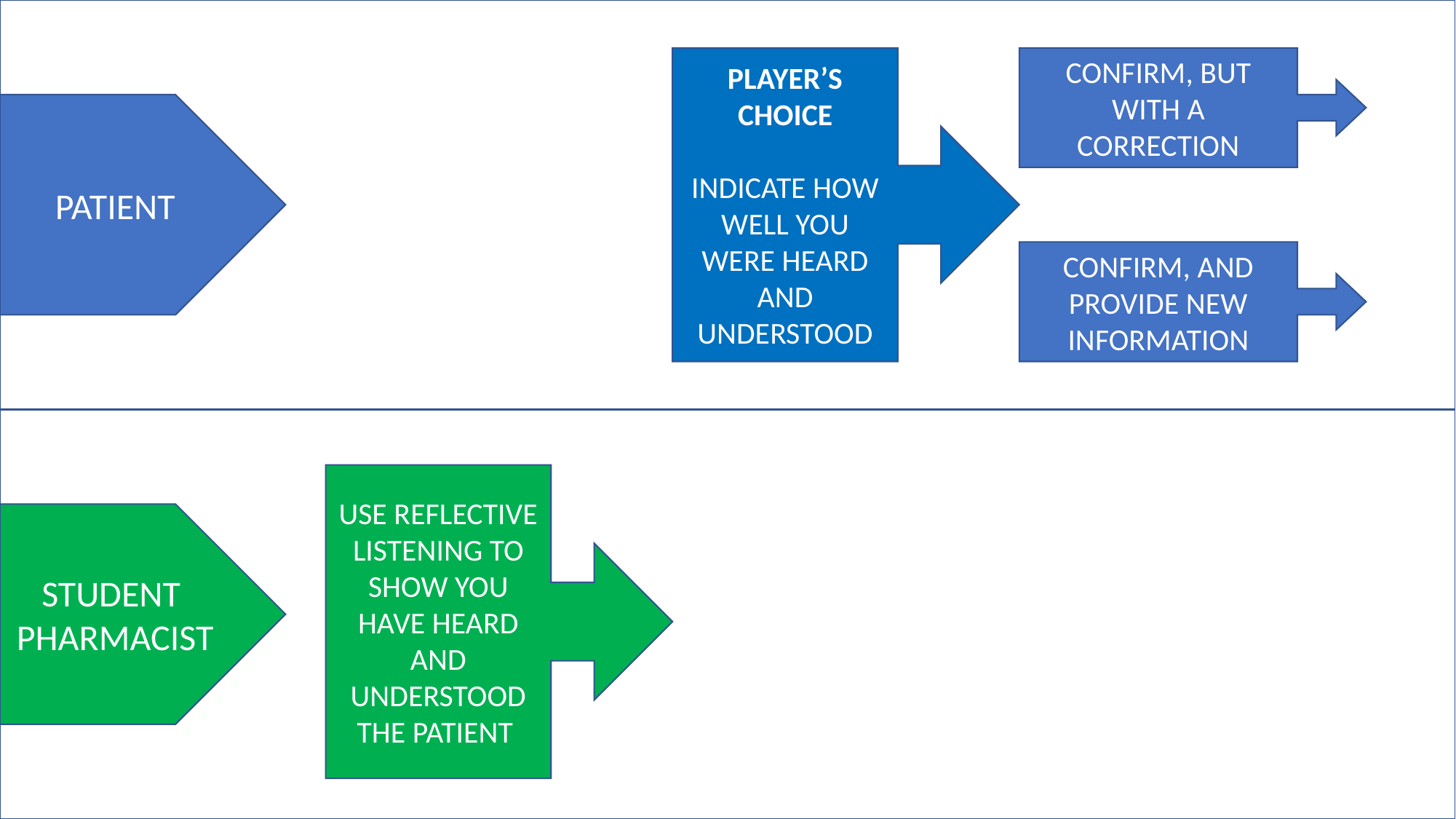

PLAYER’S CHOICE
INDICATE HOW WELL YOU WERE HEARD AND UNDERSTOOD
CONFIRM, BUT WITH A CORRECTION
PATIENT
CONFIRM, AND PROVIDE NEW INFORMATION
USE REFLECTIVE LISTENING TO SHOW YOU HAVE HEARD AND UNDERSTOOD THE PATIENT
STUDENT
PHARMACIST
17

## Slide 18
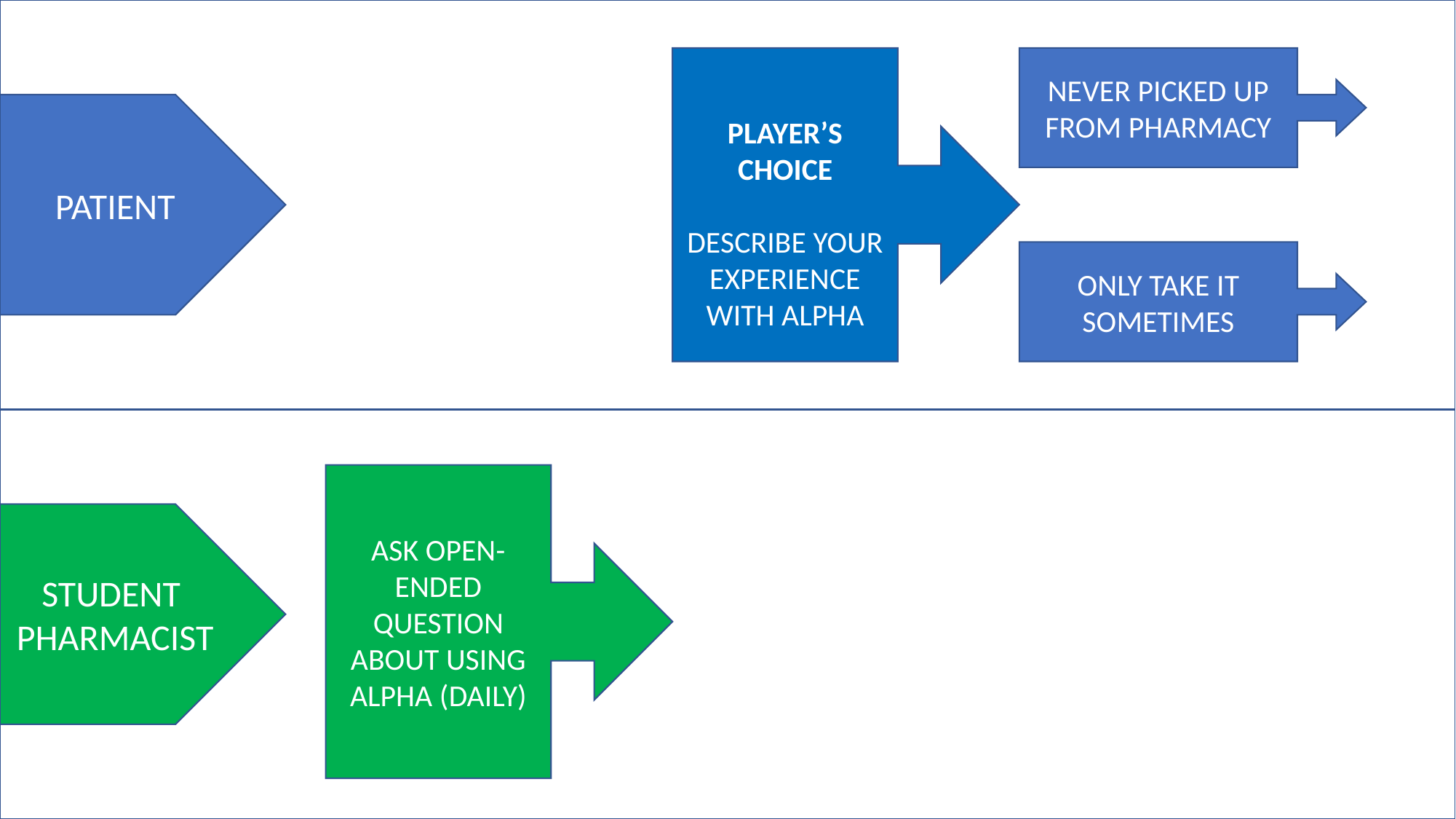

PLAYER’S CHOICE
DESCRIBE YOUR EXPERIENCE WITH ALPHA
NEVER PICKED UP FROM PHARMACY
PATIENT
ONLY TAKE IT SOMETIMES
ASK OPEN-ENDED QUESTION ABOUT USING ALPHA (DAILY)
STUDENT
PHARMACIST
18

## Slide 19
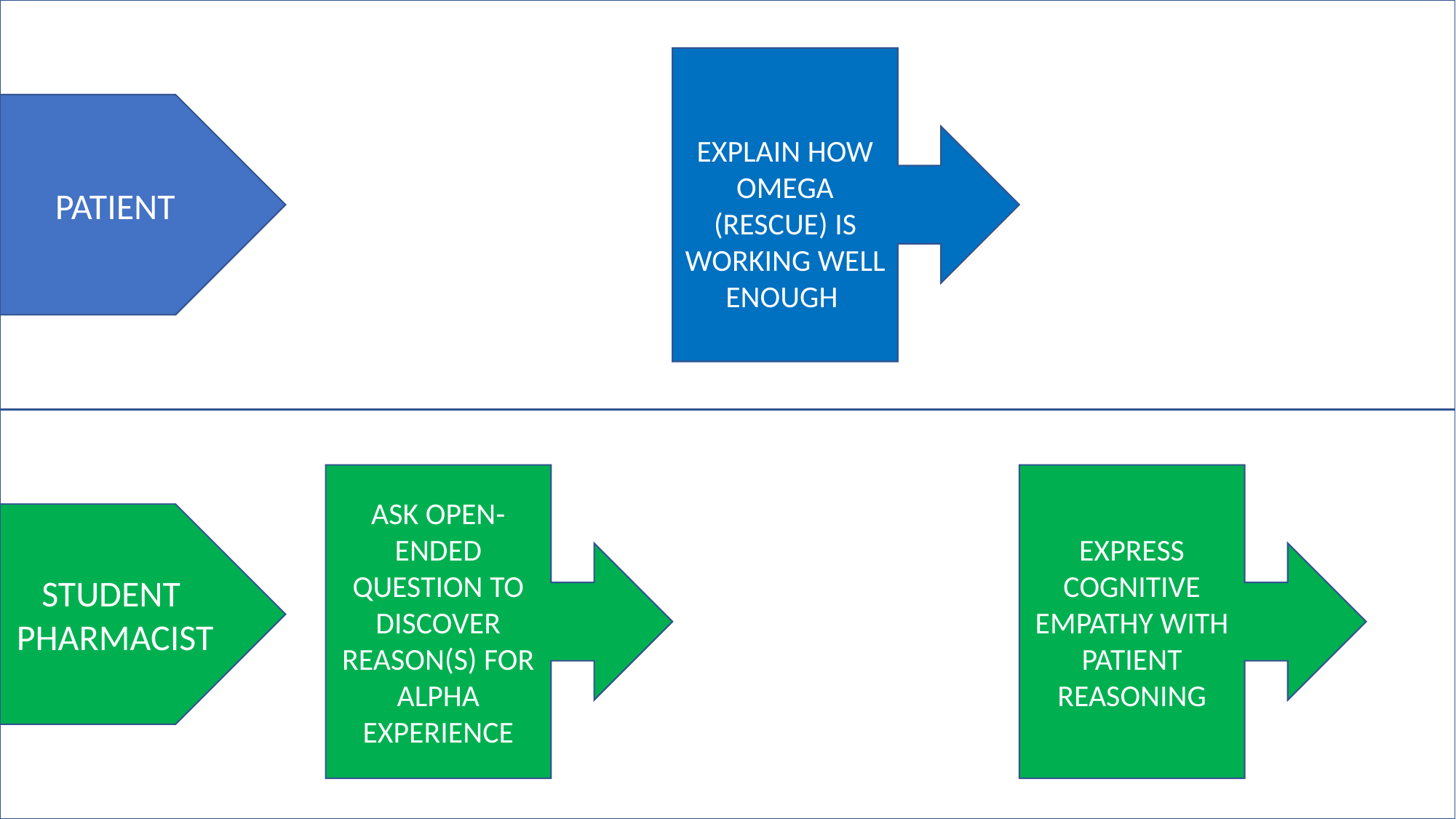

EXPLAIN HOW OMEGA (RESCUE) IS WORKING WELL ENOUGH
PATIENT
ASK OPEN-ENDED QUESTION TO DISCOVER REASON(S) FOR ALPHA EXPERIENCE
EXPRESS COGNITIVE EMPATHY WITH PATIENT REASONING
STUDENT
PHARMACIST
19

## Slide 20
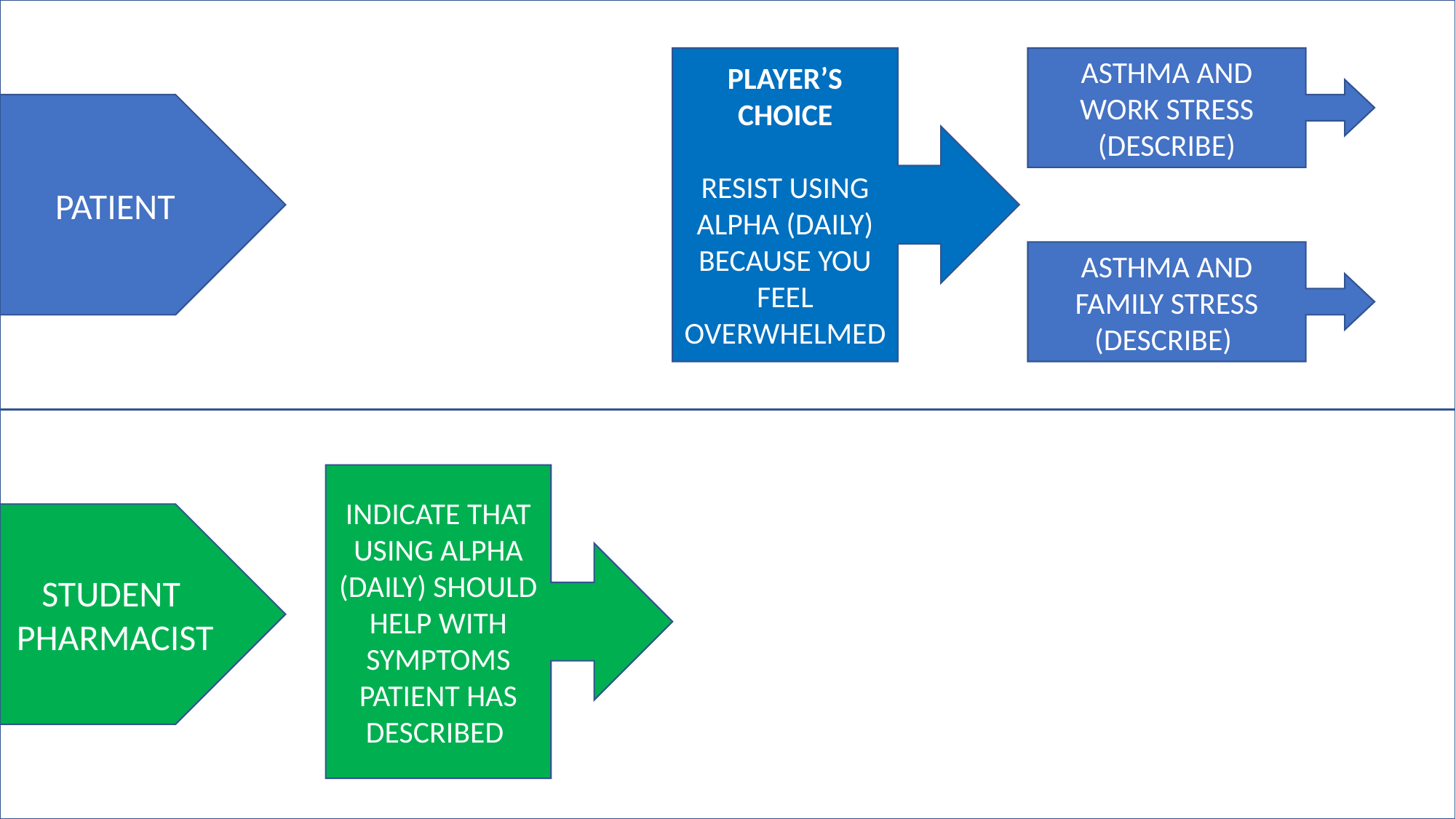

PLAYER’S CHOICE
RESIST USING ALPHA (DAILY) BECAUSE YOU FEEL OVERWHELMED
ASTHMA AND WORK STRESS (DESCRIBE)
PATIENT
ASTHMA AND FAMILY STRESS (DESCRIBE)
INDICATE THAT USING ALPHA (DAILY) SHOULD HELP WITH SYMPTOMS PATIENT HAS DESCRIBED
STUDENT
PHARMACIST
20

## Slide 21
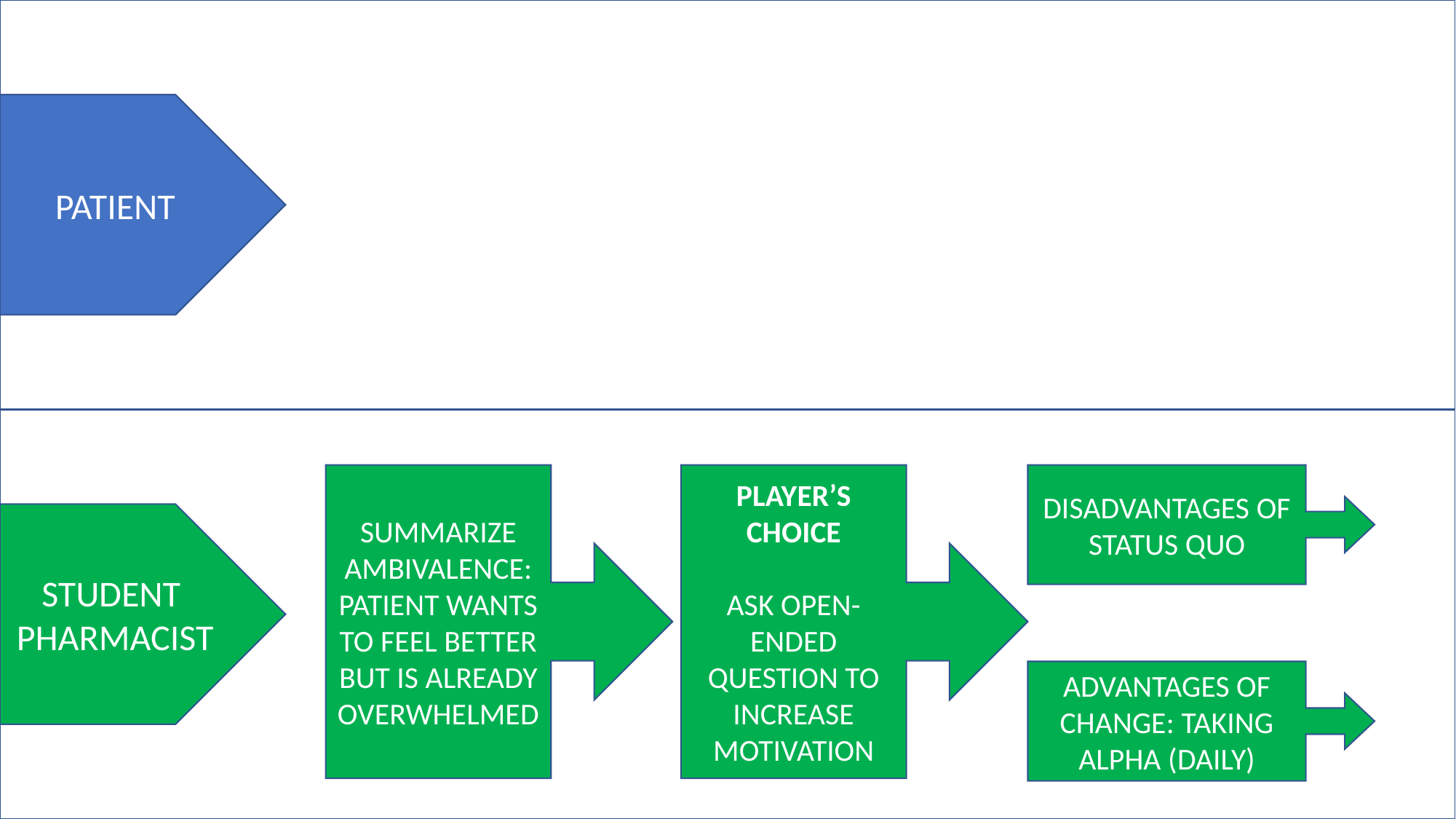

PATIENT
SUMMARIZE AMBIVALENCE: PATIENT WANTS TO FEEL BETTER BUT IS ALREADY OVERWHELMED
PLAYER’S CHOICE
ASK OPEN-ENDED QUESTION TO INCREASE MOTIVATION
DISADVANTAGES OF STATUS QUO
STUDENT
PHARMACIST
ADVANTAGES OF CHANGE: TAKING ALPHA (DAILY)
21

## Slide 22
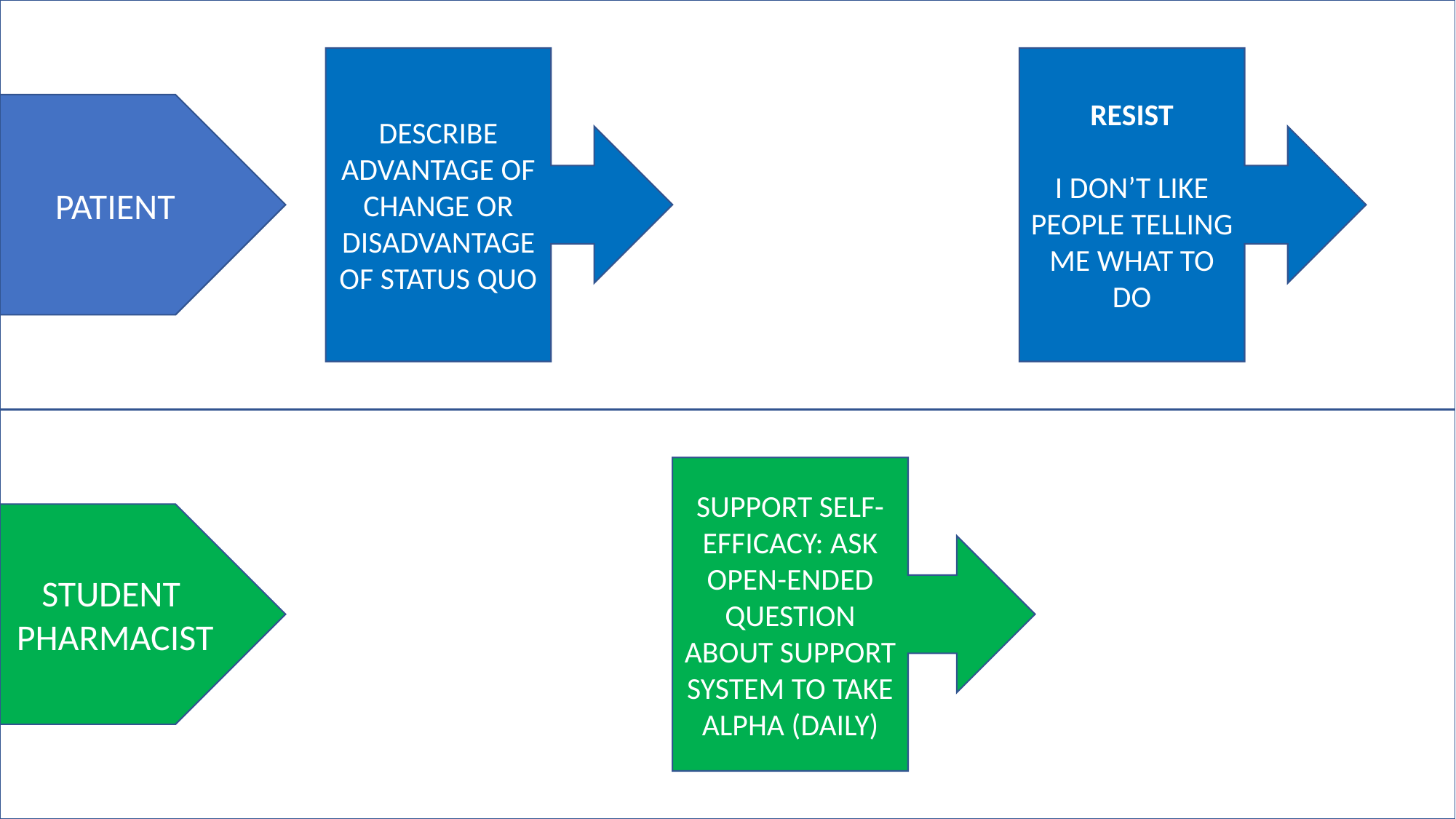

RESIST
I DON’T LIKE PEOPLE TELLING ME WHAT TO DO
DESCRIBE ADVANTAGE OF CHANGE OR DISADVANTAGEOF STATUS QUO
PATIENT
SUPPORT SELF-EFFICACY: ASK OPEN-ENDED QUESTION ABOUT SUPPORT SYSTEM TO TAKE ALPHA (DAILY)
STUDENT
PHARMACIST
22

## Slide 23
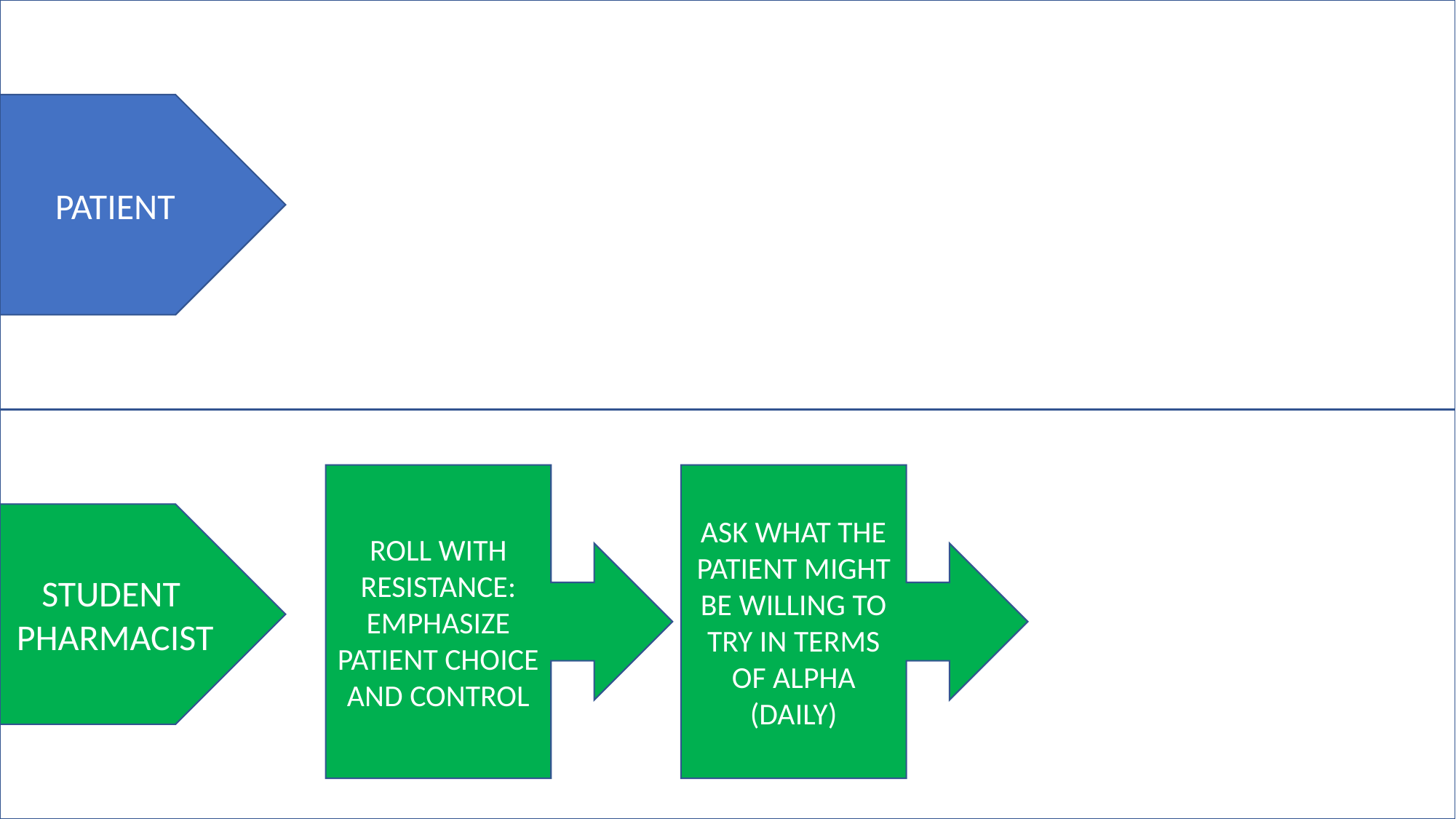

PATIENT
ROLL WITH RESISTANCE:
EMPHASIZE PATIENT CHOICE AND CONTROL
ASK WHAT THE PATIENT MIGHT BE WILLING TO TRY IN TERMS OF ALPHA (DAILY)
STUDENT
PHARMACIST
23

## Slide 24
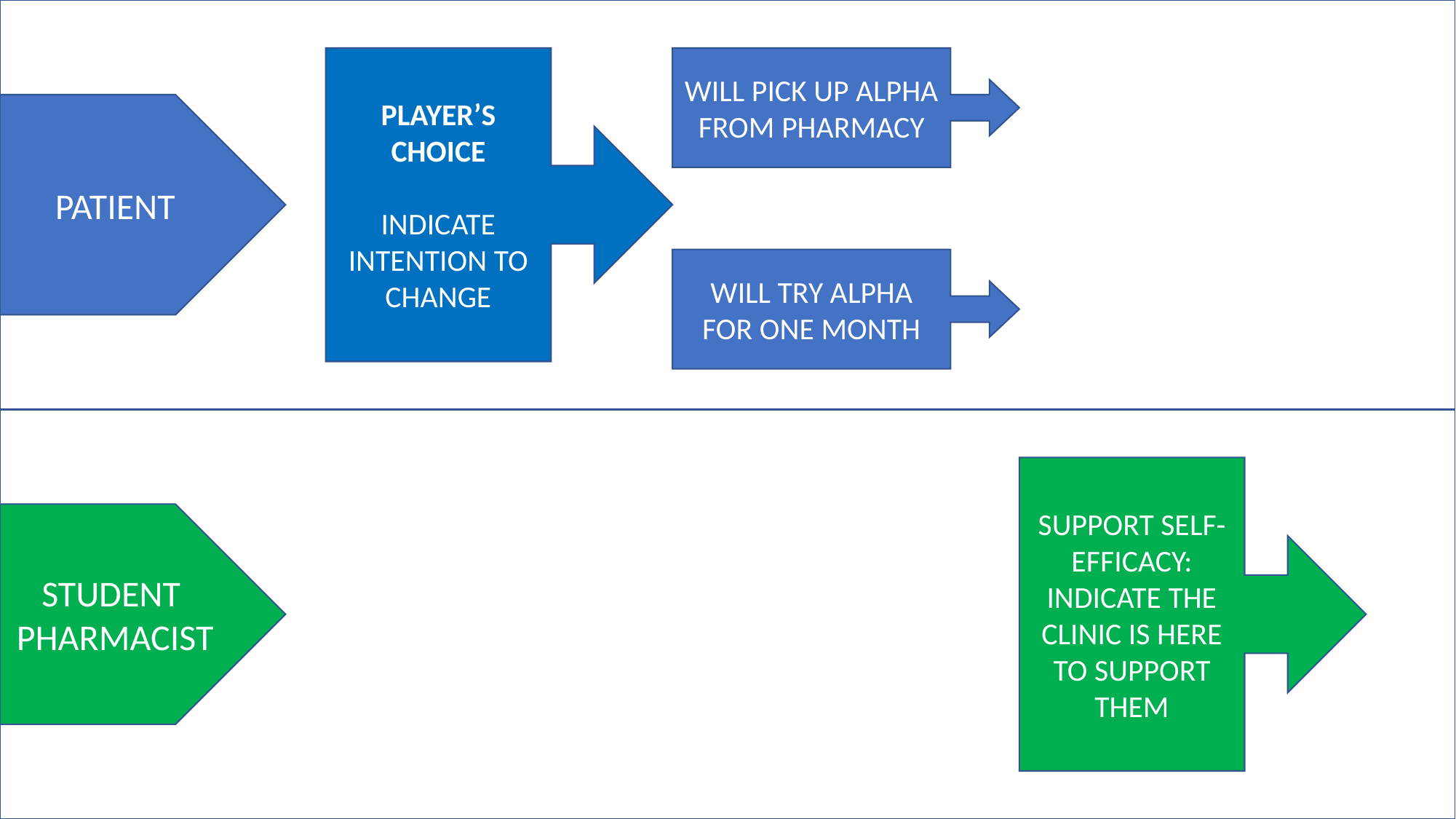

PLAYER’S CHOICE
INDICATE INTENTION TO CHANGE
WILL PICK UP ALPHA FROM PHARMACY
PATIENT
WILL TRY ALPHA FOR ONE MONTH
SUPPORT SELF-EFFICACY: INDICATE THE CLINIC IS HERE TO SUPPORT THEM
STUDENT
PHARMACIST
24

## Slide 25
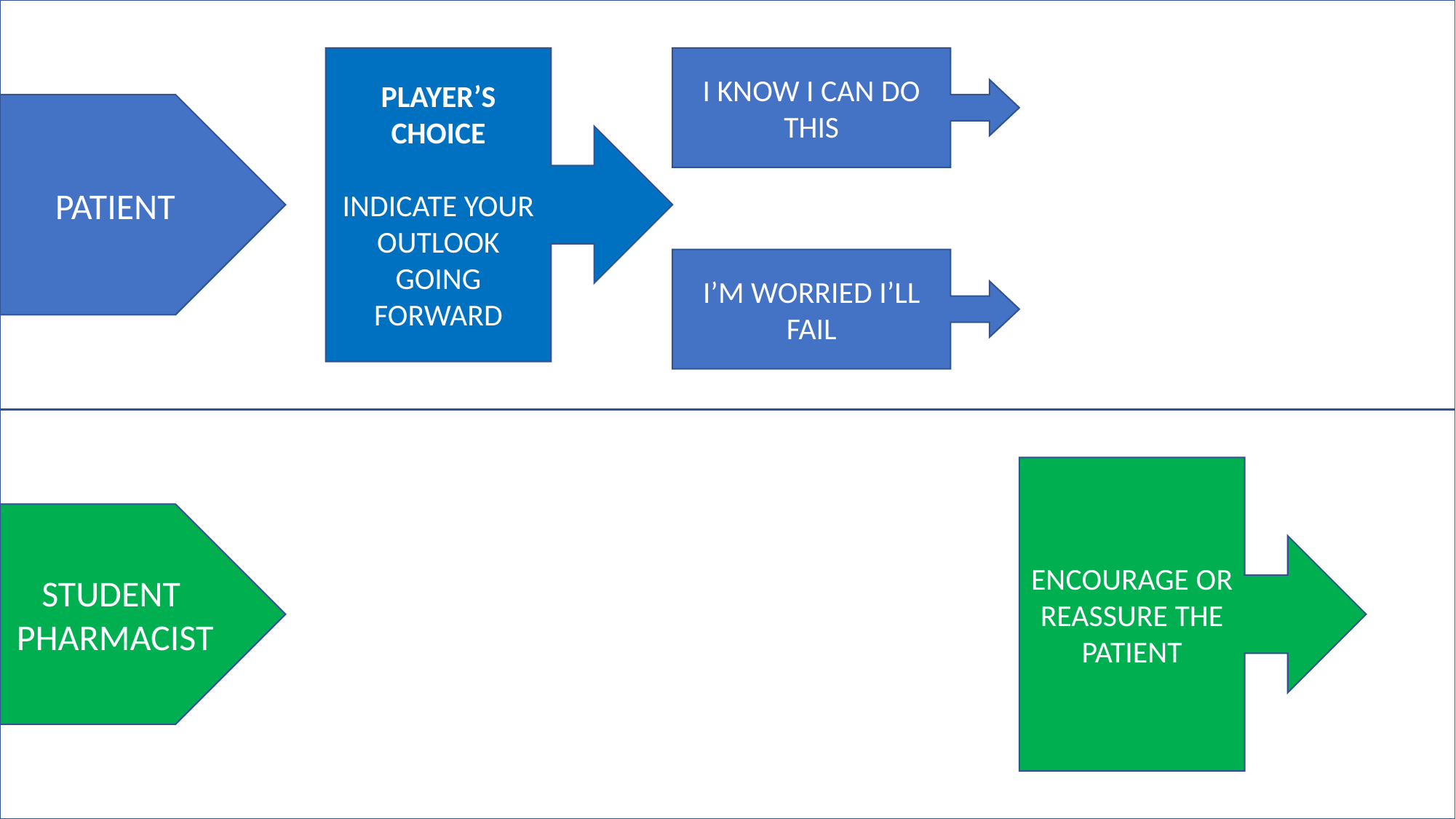

PLAYER’S CHOICE
INDICATE YOUR OUTLOOK GOING FORWARD
I KNOW I CAN DO THIS
PATIENT
I’M WORRIED I’LL FAIL
ENCOURAGE OR REASSURE THE PATIENT
STUDENT
PHARMACIST
25

## Slide 26
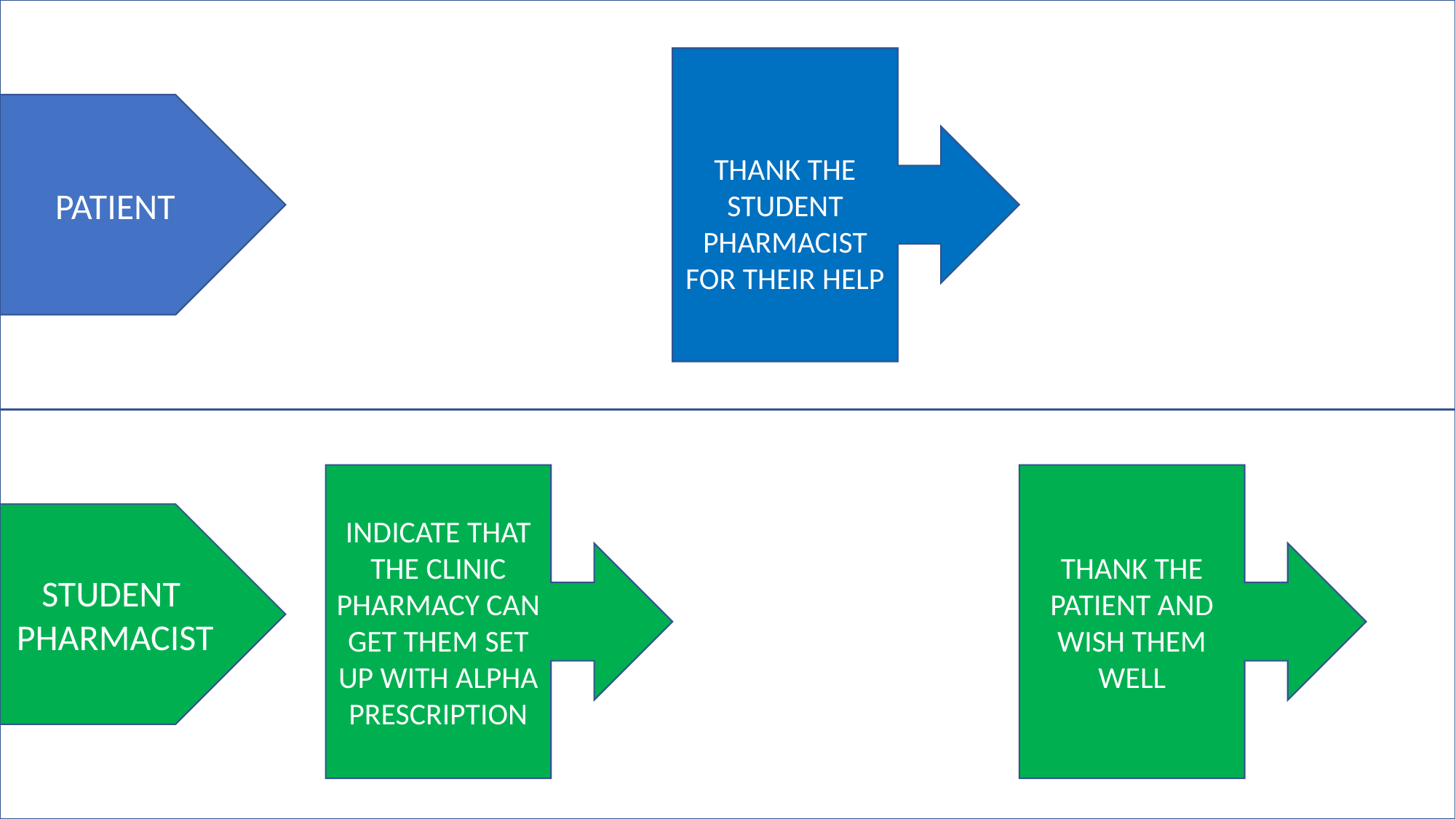

THANK THE STUDENT PHARMACIST FOR THEIR HELP
PATIENT
INDICATE THAT THE CLINIC PHARMACY CAN GET THEM SET UP WITH ALPHA PRESCRIPTION
THANK THE PATIENT AND WISH THEM WELL
STUDENT
PHARMACIST
26
