# Supplementary material for: Exploring the Impact of an Innovative Peer Role-Play Simulation to Cultivate Student Pharmacists’ Motivational Interviewing Skills
Source: Pharmacy (Basel). 2023 Jul 29;11(4):122. doi: 10.3390/pharmacy11040122 (PMC10458726; doi:10.3390/pharmacy11040122)
Supplement: Supplementary file 1 [file pharmacy-11-00122-s001.zip › Table S1-Formulation challenges in motivational interviewing.pdf]

Supplement Table 1. Formulation Challenge Assignment: Design Next Speaking Turn Using Specific MI Techniques

| MI Technique | Case Scenario | Consultation Dialogue | Examples of Students' Formulations for Speaking Turn |
|---|---|---|---|
| **Support Self-Efficacy** | At a regional hospital, a pharmacist offers tobacco cessation services to patients who are being discharged after receiving care for pulmonary and cardiovascular problems. A male patient in his 60s has been a lifelong smoker, quitting several times but ultimately returning to smoking each time. After checking up on a few other medications, the pharmacist raises the topic of the patient going home: | **1. Pharmacist: "So, it looks like you'll be heading home tomorrow, right?"**<br>2. Patient: "Yup. They said tomorrow's the day."<br>**3. Pharmacist: "So how are you feeling about it?"**<br>4. Patient: "Mostly I'm looking forward to seeing my dog. My son has been taking care of him but I know he likes me the best." (smiles, both laugh)<br>**5. Pharmacist: "So, I was also wondering how you feel about going home in terms of your smoking. Dr. Nguyen mentioned that you were hoping to quit or cut back after all of this and I may be able to help you with that." (Pause)**<br>6. Patient: "Uhh – can I be honest?"<br>**7. Pharmacist: "Of course you can."**<br>8. Patient: "I'm dying for a cigarette right now. Like, I can feel my hands shaking just thinking about smoking one when I get out of here."<br>**9. Pharmacist: "Well, that's a very normal feeling when your body is used to nicotine. Would you say you feel ready to quit smoking at this point?"**<br>10. **Patient: "I mean…I want to. I don't want to end up back here in 6 months.** But I don't know if I'm ready. I've thought I was ready a bunch of times already and failed every time, so what do I know?"<br>**11. Pharmacist: __________ Using the support self-efficacy technique of OPEN-ENDED QUESTIONS TO ELICIT OPTIMISM ABOUT CHANGE, write an effective speaking turn for the pharmacist in line 11.** | "I understand that the choice and process of quitting smoking cigarettes can be difficult. The path to recovery is usually easier if you have someone who will support you. Who can support you to stay on the right path and prevent you from smoking cigarettes when you have the need for one?" |
| **Roll with Resistance** | After a recent annual check-up with her physician, a perimenopausal woman stops by her community pharmacy to pick up some new prescriptions, including Fosamax, a medication for treating osteoporosis. The patient asks to speak privately with the pharmacist, so they sit down together in the counseling area: | **1. Pharmacist: "Alright, so how can I help you today?"**<br>2. Patient: "Well, here's the thing – I'm going to pick up the other prescriptions but not that Fosamax or whatever, the bone stuff. Nope."<br>**3. Pharmacist: "Okay is there a problem that you're having with the Fosamax? Maybe we can help."**<br>4. Patient: "No, there's no problem! But try telling that to Dr. Perkins. She's been trying to get me to take those pills for three years now and I keep telling her I don't need 'em! Maybe she'll listen to you."<br>**5. Pharmacist: "I see and is this for osteoporosis?"**<br>6. Patient: "Yeah, like I'm some frail old lady or something. I'm 45 for crying out loud!"<br>**7. Pharmacist: "Mm hm, I see where you're coming from there. Sometimes medications have a way of reminding us that we're getting older, and our bodies don't work like they used to."**<br>8. Patient: "Well, mine's working fine. Dr. Perkins ordered this bone density test a few years back and now she keeps trying to get me to take these grandma pills."<br>**9. Pharmacist: "Well, speaking as your pharmacist, if those bone density scans are showing…"** | "I completely understand that you do not want to take the medication and are not going to take it. I am not here to convince you to take something you don't want to take. I could give you some information on the medication and effects of not taking the medication just to think about if you would like." |

| | | | |
|---|---|---|---|
| | | 10. Patient: “Nope. Not doing it.”<br>**11. Pharmacist: __________ Using the roll with resistance technique of EMPHASIZING CLIENT CHOICE AND CONTROL, write an effective speaking turn for the pharmacist in line 11.** | |
| **Express Empathy** | A pharmacist is conducting a telephonic medication review with a patient who is a mother of a two-year-old daughter. During the consultation, the pharmacist asks about the patient’s use of Citalopram, a medication used to treat anxiety and depression: | 1. **Pharmacist: “So, I see a prescription here for citalopram 30 milligrams a day?”**<br>2. Patient: “Mm hmm”<br>**3. Pharmacist: “And how’s that been working out for you?”**<br>4. Patient: “Um...pretty good. I’ve been feeling a bit better...”<br>5. Patient: “But y’know to be honest I’m not talking it all the time.”<br>**6. Pharmacist: “Okay, not taking it every day?”**<br>7. Patient: “Nah not every day and sometimes I’ll cut ‘em in half or I don’t take both pills, y’know I have to take two pills, different strengths, for the 30 milligrams.”<br>**8. Pharmacist” (nods): “Mm Hmm.”**<br>9. Patient: Thing is, it does seem to help but it just makes me soooo drowsy, like I' sleepwalking or something. My daughter be like, “Mommy why you so sleepy!?”<br>10. Patient: (laughs) “And I’m just like “Sorry baby” and the thing is, I’m home alone with her all day and I need to be thinking straight.”<br>11. **Pharmacist: (laughs) “As a mom myself, I can totally relate!”**<br>12. Patient: “Y’know, and I want us to be able to do things or go to the park or whatever and it just seems like (pause) pick your poison, y’know, depression or sleepwalking.”<br>**13. Pharmacist: __________________ Write an effective speaking turn for the pharmacist in line 13 using the reflective listening technique of SUMMARIZING AMBIVALENCE** | “Yeah I understand you feel like you have a tough choice to make. On one hand, you know that by taking the prescription, you have been feeling better. On the other hand, you want a clear mind so you can fully support your daughter each day. Fortunately, I believe we can find a solution that allows you to feel better while also continuing to be a great mother. Would you like to discuss alternative treatment options?” |
| **Develop Discrepancy** | In a Family Medicine clinic, a physician has asked a pharmacist to speak with their asthmatic patient about his use of Flovent, a daily long-term control medication that helps prevent asthma attacks. The patient also has a prescription for Albuterol, a rescue inhaler to be used in emergencies. | **1. Pharmacist: “How’s the asthma been going lately?”**<br>2. Patient: “Pretty smooth I’d say.”<br>**3. Pharmacist: “Yeah? That’s great to hear.”**<br>4. Patient: “Mm hmm. Whenever it flairs up I’ve got that Albuterol that you folks gave me last year. I swear, at least once or twice a day, I take it and tell myself, ‘This stuff is a miracle!’ It scares me to think what I’d do if I didn’t have it on hand. A couple of times I forgot it and had to drive back home to grab it.”<br>**5. Pharmacist: “I’m really glad the Albuterol is working for you. How about the Flovent? Have you noticed any improvement with that?”**<br>6. Patient: “Uhhh, of the Flovent. Yeah, I don’t really bother with that one. That’s the daily one, right? Dr. Denard’s been on me about that one (laughs) but | “Well I’m glad to hear that the albuterol is working for the attacks, but how would it feel knowing that you wouldn’t have to worry about asthma attacks as much if you started taking Flovent?” |

| | | | |
|---|---|---|---|
| | The physician and pharmacist agree that the patient would benefit from adhering more consistently to Flovent, the daily control medication: | honestly the Albuterol seems to be enough for me. I'd rather not be taking something every day if I don't need to y'know."<br>**7. Pharmacist: "Well, I certainly understand that."**<br>8. Patient: "And I can usually tell if an attack is coming on, so…y'know I think the Albuterol is all I need."<br>**9. Pharmacist: ______________ Using the developing discrepancy technique of OPEN-ENDED QUESTIONS ABOUT DISADVANTAGES OF STATUS QUO, write an effective speaking turn for the pharmacist in line 9, or Using the developing discrepancy technique of OPEN-ENDED QUESTIONS ABOUT ADVANTAGES OF CHANGE, write an effective speaking turn for the pharmacist in line 9.** | |
