# Supplementary material for: Exploring the Impact of an Innovative Peer Role-Play Simulation to Cultivate Student Pharmacists’ Motivational Interviewing Skills
Source: Pharmacy (Basel). 2023 Jul 29;11(4):122. doi: 10.3390/pharmacy11040122 (PMC10458726; doi:10.3390/pharmacy11040122)
Supplement: Supplementary file 1 [file pharmacy-11-00122-s001.zip › Table S2-MI role-play grading rubric.pdf]

Motivational Interviewing Peer Role-play Grading Rubric

	Ratings Point Allocation					
**Criteria**	3	2	1	0.5	0	**Total Points**
Student Pharmacist: Employs principle of **patient autonomy**. Reassures patient that they are in control (slides 13 and 23)	X	X	**Fully Achieved** Consistently emphasizes patient autonomy	**Partially Achieved** Sometimes challenges patient autonomy or exerts authority	**Not achieved** Consistently challenges patient autonomy or exerts authority	1
Student Pharmacist: **Expresses empathy.** Expresses emotional and/or cognitive empathy using paraphrasing (slides 12, 15, 17, 19); Summarizes ambivalence using paraphrasing (slide 21)	**Fully Achieved** Consistently expresses empathy using multiple counseling practices	**Mostly Achieved** In most cases, expresses empathy effectively using multiple counseling practices	**Partially Achieved** Frequently misses opportunities to express empathy, struggles with one or more counseling practices, or uses formulations that are mildly judgmental of patient's choices and circumstances	X	**Not Achieved** Consistently misses opportunities to express empathy or uses formulations that are judgmental of patient's choices and circumstances	3
Student Pharmacist: **Develops discrepancy.** Provides opportunity for the patient to talk about distance between current state and goal state (slide 21)	X	X	**Fully Achieved** Consistently helps patient develop discrepancy when opportunities arise	**Partially Achieved** Helps to develop discrepancy in some ways, but uses authority to assert that the goal state is far away or uses formulation(s) that are not open-ended	**Not Achieved** Does not help patient develop discrepancy	1
Student Pharmacist: Enhances patient's **self-efficacy**. Helps patient build confidence through change talk, including possible sources of	X	**Fully Achieved** Consistently creates opportunities for the patient to engage in change talk that builds	**Partially Achieved** Enhances self efficacy in most opportunities but doesn't consistently encourage the patient to be the one to voice their own confidence	X	**Not Achieved** Consistently misses opportunities to enhance patient's self-efficacy	2

social/professional support (slides 22, 23, 24, 25)		confidence and identifies sources of support				
Student Pharmacist: **Rolls with resistance** When faced with patient resistance, is non-confrontational, shifts the topic, and/or emphasizes patient autonomy (slide 13 and 23)	X	X	**Fully Achieved** Consistently avoids confrontation and/or redirects the conversation when resistance emerges; guides patient without asserting authority/expertise	**Partially Achieved** Attempts to avoid confrontation or debate, but uses formulations that could invite patient to resist even more (dig in their heels)	**Not Achieved** Is confrontational or attempts to argue, debate, or correct the patient excessively	1
Patient: **Prepares well** to describe symptoms and life circumstances with accuracy and realism	X	X	**Fully Achieved** Appears familiar with the flow of the timeline and is fully prepared to describe asthma symptoms and life circumstances; speaks conversationally, avoiding excessively short speaking turns	**Partially Achieved** Appears moderately well-prepared but misses some key speaking turns in the timeline and/or doesn't describe symptoms accurately or life circumstances with realism/authenticity	**Not Achieved** Appears poorly prepared to provide appropriate portrayal of the patient; performance hinders partner's ability to practice Motivational Interviewing principles	1
Patient: **Presents a balanced challenge** for partner using reasonable resistance and change talk	X	X	**Fully Achieved** Consistently provides a reasonable and balanced challenge, resisting appropriately as directed and engaging in change talk when student pharmacist uses effective Motivational Interviewing practices	**Partially Achieved** Mostly provides a balanced challenge for the student pharmacist but is occasionally excessively difficult or excessively easy	**Not Achieved** Provides an excessively difficult or excessively easy encounter; performance hinders partner's ability to practice Motivational Interviewing principles	1
Total Points						10
